# Extracellular vesicles in wound healing and scar formation: molecular regulation of macrophages, fibroblasts, and their crosstalk

**DOI:** 10.3389/fcell.2026.1917543

**Published:** 2026-09-11

**Authors:** Tianqi Liu, Yuxin Liu, Yangdan Liu, Dongsheng Wen, Jiaming Sun, Yifan Zhang, Ya Gao, Danning Zheng

**Affiliations:** Department of Plastic & Reconstructive Surgery, Shanghai Ninth People’s Hospital, School of Medicine, Shanghai Jiao Tong University, Shanghai, China

**Keywords:** extracellular vesicles, fibroblasts, macrophage–fibroblast crosstalk, macrophages, pathological scarring, wound healing

## Abstract

Extracellular vesicles (EVs) mediate communication between immune and stromal cells during cutaneous repair by transferring proteins, lipids, nucleic acids, and other bioactive components. This review examines their effects on macrophage inflammatory and stress-adaptation programs, fibroblast activation and fate, and macrophage–fibroblast crosstalk in wound healing and pathological scarring. In chronic and metabolically impaired wounds, stem- and stromal-cell EVs commonly reduce persistent inflammation, improve macrophage autophagy and redox balance, and support fibroblast migration, proliferation, survival, and provisional matrix formation. In scar models, some EV preparations attenuate sustained profibrotic and mechanotransduction signaling, whereas EVs from diseased or microenvironmentally conditioned cells can prolong inflammation, maintain myofibroblast activity, and increase collagen deposition. Direct evidence for EV-mediated crosstalk is less extensive than evidence for effects on either cell type alone but supports communication in both directions. Macrophage-derived EVs alter fibroblast metabolism, growth signaling, autophagy, and extracellular matrix (ECM) production through long noncoding RNAs, microRNAs, and chemokines. Fibroblast-derived EVs can coordinate phase-specific macrophage responses during repair, whereas EVs from diabetic ulcers or fibrotic skin may impair macrophage autophagy, activate inflammasome signaling, and reinforce profibrotic feedback. EV activity therefore depends on the state of both donor and recipient cells, the local matrix and metabolic environment, dose, and timing. Particular emphasis is placed on reciprocal EV-mediated macrophage–fibroblast signaling, an aspect of cutaneous repair that remains insufficiently synthesized despite increasing mechanistic evidence. Clinical translation will require causal validation of EV transfer, standardized product characterization, and long-term assessment of wound closure and scar quality.

## 1 Introduction

Extracellular vesicles (EVs) are lipid-bilayer-delimited particles released by cells and can transfer proteins, lipids, messenger RNAs, microRNAs (miRNAs), and other bioactive molecules between cells (Cook and Li, 2025). They arise through endosomal, plasma-membrane budding, and apoptotic pathways, although particle size alone does not define a specific biogenetic origin. The Minimal Information for Studies of Extracellular Vesicles 2023 (MISEV 2023) guidelines therefore recommend the generic term EV when biogenesis has not been demonstrated and discourage the use of terms such as “exosome” solely on the basis of size (Welsh et al., 2024). This review consequently uses EV as the default term while retaining the terminology of individual studies where needed.

Owing to their disease-associated alterations and functional roles in pathogenesis, EVs are increasingly recognized as promising biomarkers for diagnostic and prognostic applications (Sun and Chang, 2024). Additionally, their low immunogenicity and capacity for engineered cargo loading position EVs as attractive platforms for biopharmaceutical development, enhancing the efficacy and delivery of therapeutic agents (Ebrahimi et al., 2024). However, translation depends on rigorous definition of the source material, standardized separation and characterization, and potency assays linked to the intended biological activity (Ebr et al., 2024).

Cutaneous repair proceeds through overlapping phases of hemostasis, inflammation, proliferation, and remodeling, during which macrophages coordinate pathogen clearance, debris removal, inflammatory resolution, and growth-factor production (Liu et al., 2025a; Hassanshahi et al., 2022). Fibroblasts restore the extracellular matrix (ECM), support granulation tissue, and generate contractile myofibroblasts (Talbott et al., 2022). These responses support closure when they are appropriately timed and resolved. Persistent or dysregulated inflammation contributes to chronic wound failure (Hesketh et al., 2017), and infection adds further barriers to repair in diabetic foot wounds (Turzańska et al., 2023). Prolonged fibroblast activation and impaired myofibroblast clearance promote hypertrophic scars and keloids (Younesi et al., 2024; Ma et al., 2023).

Recent reviews have addressed EV-based wound repair broadly (Eerdekens et al., 2025). Other syntheses have focused specifically on macrophage-derived EVs in diabetic wounds (Shi et al., 2025). Fibroblast-derived EVs have also been reviewed as regulators of wound repair (Sadeghi et al., 2025). EV-mediated cell–cell communication has additionally been reviewed in keloids and hypertrophic scars (Lu et al., 2026). A synthesis centered specifically on reciprocal macrophage–fibroblast EV signaling across both wound healing and pathological scarring, however, remains lacking. By integrating evidence from both directions of communication with the cellular and temporal contexts in which these interactions occur, this review aims to provide a distinct conceptual framework for understanding how EV-mediated immune–stromal crosstalk shapes repair and fibrosis.

## 2 EV-mediated regulation of macrophages in wound healing and scar formation

### 2.1 Macrophage heterogeneity and stage-dependent functions

The classical M1/M2 framework remains useful as shorthand for inflammatory and repair-associated macrophage programs, but these states are neither fixed nor mutually exclusive. Human wound macrophages occupy a continuum shaped by ontogeny, local cytokines, metabolic conditions, phagocytic activity, and time after injury (Liu et al., 2025a; Murray et al., 2014). Here, M1-like and M2-like denote operational states rather than stable phenotypes.

Early inflammatory macrophage activity supports antimicrobial defense and removal of damaged tissue, whereas later resolving and repair-associated programs promote angiogenesis, fibroblast recruitment, and matrix restoration (Hassanshahi et al., 2022; Hesketh et al., 2017). Persistent inflammatory activity is characteristic of chronic wounds (Turzańska et al., 2023), while prolonged repair-associated macrophage signaling can also sustain fibroblast activation and matrix deposition in pathological scars (Xu et al., 2020). Thus, the tissue consequence of an EV-induced macrophage response depends on both its functional program and its timing within repair.

### 2.2 EVs regulate macrophage function

#### 2.2.1 Evidence in wound healing

Macrophage responses to EVs reflect interacting inflammatory and repair programs rather than a single polarization switch. In a pressure-ulcer model, miR-21-5p-enriched EVs from adipose-derived stem cells (ADSC-EVs) increased repair-associated macrophage markers and suppressed nuclear factor kappa B (NF-κB) signaling (Su et al., 2025), whereas ADSC-EV-associated circRps5 shifted macrophage markers through miR-124–3p in diabetic wounds, although the downstream protein target was not resolved (Yin and Shen, 2024).

Janus kinase/signal transducer and activator of transcription (JAK/STAT) signaling provides another context-dependent node. Epidermal stem cell EV-associated miR-203a-3p modulated suppressor of cytokine signaling 3 (SOCS3)/JAK2/STAT3 signaling in diabetic wounds (Yang et al., 2023), while miR-1246-enriched EVs from stem cells isolated from human exfoliated deciduous teeth activated protein kinase B (AKT), extracellular signal-regulated kinase 1/2 (ERK1/2), and STAT3, increased macrophage autophagy, and improved healing in a lipopolysaccharide-associated wound model (Xie et al., 2022). ADSC-derived apoptotic bodies carrying miR-20a-5p likewise altered JAK/STAT-associated macrophage responses in diabetic wounds (Zhou et al., 2022).

EV regulation of macrophages also extends beyond polarization markers. Extracellular vesicles from human umbilical cord mesenchymal stromal cells (hUCMSC-EVs) increased arginase 1 (ARG1), reduced inflammatory mediators, and improved angiogenesis and collagen deposition in diabetic wounds (Teng et al., 2022), consistent with a shift toward repair-associated function without requiring a stable M2-like state. Keratinocyte EV-associated metastasis-associated lung adenocarcinoma transcript 1 (MALAT1) relieved miR-1914-3p-mediated repression of milk fat globule-EGF factor 8 (MFG-E8) and increased repair-associated macrophage markers (Kuang et al., 2023). In parallel, apoptotic hUCMSC-EVs reduced high-glucose-induced macrophage pyroptosis and oxidative stress in type 2 diabetic wounds (Wang et al., 2023a). Together, these findings implicate autophagy, redox adaptation, inflammatory cell death, and cytokine signaling as functionally relevant EV-responsive macrophage programs.

Comparison across wound-healing studies remains limited by heterogeneous macrophage-state definitions, EV doses, treatment schedules, and endpoints. Marker changes are most informative when accompanied by functional measures such as cytokine output, phagocytosis or efferocytosis, redox control, autophagy, and effects on neighboring cells.

#### 2.2.2 Evidence in scar formation

Scar studies contain fewer direct EV data than the wound-healing literature. An M2-like shift may help resolve excessive inflammation, but it is not inherently antifibrotic. Repair-associated macrophages can maintain fibroblast proliferation and matrix production through transforming growth factor-beta 1 (TGF-β1), platelet-derived growth factor (PDGF), and amphiregulin-associated signaling (Ueshima et al., 2019; Aran et al., 2019; Minutti et al., 2019). A macrophage program that supports closure early in repair may therefore promote fibrosis if it persists.

In hypertrophic-scar models, EVs released by M2-like macrophages and enriched in C-X-C motif chemokine ligand 2 (CXCL2) activated C-X-C chemokine receptor 7 (CXCR7)/mechanistic target of rapamycin (mTOR) signaling in hypertrophic-scar fibroblasts, increasing proliferation, migration, autophagy, and collagen deposition (Shi et al., 2024). This cargo–receptor pathway contrasts with wound studies that infer benefit mainly from macrophage markers and shows why an M2-like label alone cannot predict tissue outcome.

Scar and chronic-wound studies usually differ in model, EV preparation, treatment schedule, and endpoint, and few follow one intervention across inflammatory, proliferative, and remodeling phases. The point at which a repair-associated macrophage response becomes fibrogenic therefore remains poorly defined. A shift from early benefit to later fibrosis is biologically plausible but has rarely been followed within the same experimental system.

**FIGURE 1:**
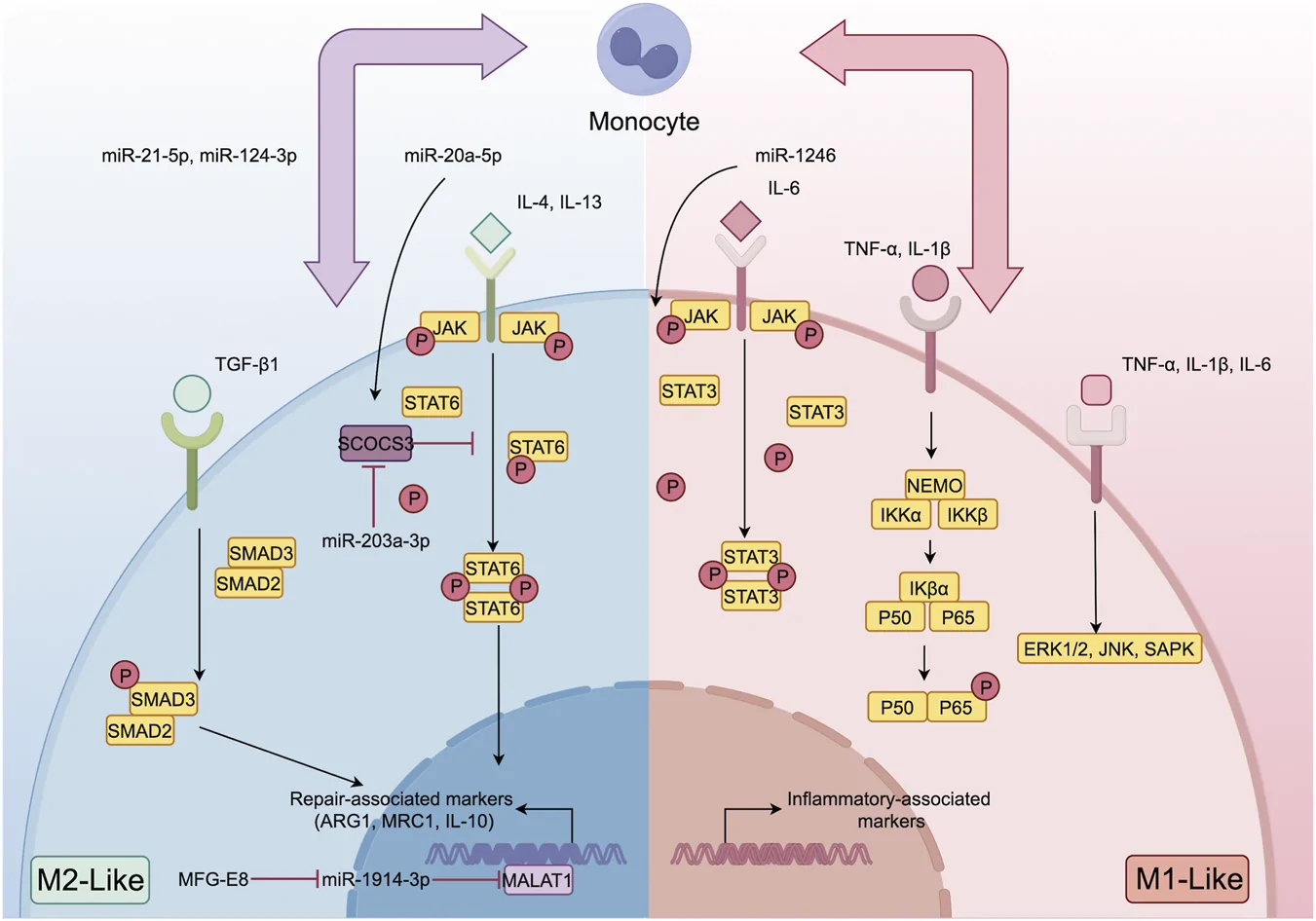
EV-mediated regulation of macrophages in wound healing and scar formation. EV-associated miR-20a-5p and miR-203a-3p modulate JAK/STAT-related macrophage programs, whereas miR-1246-enriched EVs from stem cells isolated from human exfoliated deciduous teeth promote macrophage autophagy through AKT, ERK1/2, and STAT3 signaling. Keratinocyte EV-associated MALAT1 relieves miR-1914-3p-mediated repression of MFG-E8. TGF-β1/SMAD and NF-κB/MAPK are shown as broader regulators of macrophage-state transitions. Abbreviations: AKT, protein kinase B; ERK, extracellular signal-regulated kinase; IKK, IκB kinase; JAK, Janus kinase; MALAT1, metastasis-associated lung adenocarcinoma transcript 1; MAPK, mitogen-activated protein kinase; MFG-E8, milk fat globule-EGF factor 8; NEMO, NF-κB essential modulator; NF-κB, nuclear factor kappa B; SOCS3, suppressor of cytokine signaling 3; STAT, signal transducer and activator of transcription; TGF-β1, transforming growth factor-beta 1. Created with Figdraw.

Collectively, these findings indicate that EVs regulate macrophages and related inflammatory programs through multiple cargos and signaling pathways, thereby shaping wound healing and scar outcomes (Figure 1).

## 3 EV-mediated control of fibroblast dynamics in wound healing and scar formation

### 3.1 Fibroblast heterogeneity and stage-dependent functions

Skin fibroblasts are anatomically and transcriptionally heterogeneous, and their distribution changes across wound regions and repair stages. A spatiotemporal single-cell roadmap of human skin wounds identified stage-dependent fibroblast programs during inflammation, proliferation, and remodeling (Liu et al., 2025a).

In a murine stented-wound model, integrated single-cell transcriptomic and chromatin-accessibility profiling with spatial transcriptomics showed that resident, mechanoresponsive fibroblast populations expand polyclonally after injury, occupy spatially distinct wound regions, and follow different migration, proliferation, and differentiation trajectories (Foster et al., 2021). In diabetic foot ulcers, single-cell profiling combined with spatial transcriptomics identified a healing-associated fibroblast population enriched for MMP1, MMP3, MMP11, HIF1A, CHI3L1, and TNFAIP6 that localized preferentially to the wound bed rather than the wound edge or unwounded skin (Theocharidis et al., 2022). Together, these studies place fibroblast activation within defined temporal and anatomical compartments rather than a single uniform wound population.

A broader spatial atlas of more than 350,000 adult human skin fibroblasts subsequently resolved immune-associated and disease-specific stromal states. CCL19^+^CD74^+^HLA-DRA^+^ fibroblastic reticular cell-like fibroblasts localized to superficial perivascular immune niches, whereas IL11^+^MMP1^+^CXCL8^+^IL7R^+^ inflammatory myofibroblasts were enriched in early wounds and inflammatory skin diseases associated with scarring risk. In time-resolved human wounds, this inflammatory myofibroblast state was detectable on day 1 and predominated by day 7, whereas F7 myofibroblasts characterized by stronger ECM- and TGF-β-associated programs predominated by day 30 (Steele et al., 2025). Spatial transcriptomics of keloids has likewise shown that disease-associated fibroblasts are preferentially enriched in deeper keloid regions around endothelial transcriptional domains, indicating marked intralesional compartmentalization of profibrotic stromal states (Shim et al., 2022). Most EV studies still rely on bulk dermal, hypertrophic-scar, or keloid fibroblast cultures and are therefore not designed to resolve state-specific differences in EV production or responsiveness among these wound- and scar-associated fibroblast populations.

### 3.2 EVs regulate fibroblast proliferation, activation, and extracellular matrix remodeling

#### 3.2.1 Evidence in wound healing

Fibroblast activation during normal repair is graded. Quiescent fibroblasts first acquire migratory, proliferative, and matrix-producing functions; only a subset develops a strongly contractile myofibroblast phenotype, and the persistence of these activated cells influences whether repair resolves or progresses toward scar formation (Younesi et al., 2024; Shook et al., 2018). EV studies often use “activation” to describe the stage, which can blur the distinction between restoration of deficient repair and induction of a phenotype that persists after closure.

During the proliferative phase, several EV preparations restore fibroblast recruitment and expansion in impaired wounds. Bone marrow mesenchymal stromal cell EV-associated miR-542–3p increased fibroblast motility (Xiong et al., 2023), and miR-126-3p-enriched ADSC-EVs enhanced fibroblast migration and angiogenic activity in full-thickness defects (Ma et al., 2022). Hair-follicle stromal cell EVs protected human dermal fibroblasts from hyperglycemia-associated oxidative and cytotoxic injury (Las Heras et al., 2022). In a protein-centered example, cord blood plasma EV preparations enriched in heat shock protein 72 (HSP72) and phosphatidylinositol transfer protein supported fibroblast function, angiogenesis, and repair-associated macrophage responses (Kim et al., 2021). These studies primarily address restoration of an inadequate early reparative response rather than long-term scar remodeling.

TGF-β/SMAD signaling is central to the distinction between productive and persistent fibroblast activation (Zhao et al., 2020). In diabetic wounds, ADSC-EVs carrying miR-204–5p (Song P. et al., 2025) or miR-128-1-5p (Liang et al., 2024) reduced TGF-β/SMAD activity while improving repair. Human umbilical cord blood mesenchymal stromal cell (MSC)-derived EVs carrying miR-21–5p and miR-125b-5p targeted transforming growth factor-beta receptor 2 (TGFBR2) and receptor 1 (TGFBR1), respectively, thereby attenuating TGF-β1-induced myofibroblast differentiation and limiting scar formation (Zhang et al., 2021a). These findings are most consistent with modulation of excessive or prolonged TGF-β signaling rather than complete pathway suppression.

Non-SMAD pathways regulate complementary aspects of fibroblast activity. Surface-associated heat shock protein 90 (HSP90) on ADSC-EVs activated low-density lipoprotein receptor-related protein 1 (LRP1)/AKT signaling in diabetic wounds (Ren et al., 2022), whereas another ADSC-EV preparation enhanced dermal fibroblast repair through Wnt/β-catenin signaling (Li et al., 2022). EVs from dermal papilla cells also engaged Wnt/β-catenin during hair-follicle regeneration after injury (Shang et al., 2024). EVs from mouse breast carcinoma cells carrying miR-126a-3p activated phosphoinositide 3-kinase (PI3K)/AKT signaling and enhanced fibroblast migration, proliferation, and angiogenesis in a wound model (Zhang et al., 2024). Although mechanistically informative, tumor-derived EVs have limited therapeutic relevance because of their pathological origin. EVs from epidermal stem cells were linked to protein kinase N1, cyclin, and tumor necrosis factor-related signaling in fibroblasts (Kang et al., 2024), while mast-cell EV-associated semaphorin 7A increased fibroblast proliferation, fibronectin, and type I collagen (Cho et al., 2025). Saliva-derived EVs increased glycolytic flux and matrix metalloproteinase output, linking metabolic adaptation to extracellular matrix turnover (Song S. et al., 2025). Because most of these studies focus on short-term proliferation or collagen assays, their implications for mature matrix organization remain unclear.

EVs also preserve fibroblast function in hostile wound environments. ADSC-EVs restored proliferation and migration under high-glucose conditions (Yang et al., 2024), and protection from oxidative injury was linked to the Kelch-like ECH-associated protein 1/nuclear factor erythroid 2-related factor 2 axis (Tian et al., 2025). In irradiated skin, ADSC-EVs reduced oxidative stress and proteins associated with apoptosis and pyroptosis, including BCL2-associated X protein (BAX), caspase-3, gasdermin D (GSDMD), and caspase-1 (Liu et al., 2025b). Apple-derived nanovesicles (ADNVs) reduced Toll-like receptor 4 activity and NF-κB signaling, increased type I collagen production, and suppressed matrix metalloproteinases 1, 8, and 9 in an aging-skin model (Trentini et al., 2022). As plant-derived nanoparticles, ADNVs differ from mammalian EVs in composition, uptake, nomenclature, and regulatory considerations.

#### 3.2.2 Evidence in scar formation

In pathological scars, fibroblast activation is already established; the therapeutic challenge is to terminate persistent matrix-producing and contractile programs. EVs released by hypertrophic-scar fibroblasts activated SMAD and transforming growth factor-beta-activated kinase 1 (TAK1) signaling in normal dermal fibroblasts and increased proliferation and migration, indicating that diseased fibroblast EVs can propagate a profibrotic phenotype (Cui et al., 2022).

By contrast, extracellular vesicles from mesenchymal stromal cells (MSC-EVs) have been used to interrupt several points in the TGF-β/SMAD network. ADSC-EV-associated miR-125b-5p suppressed SMAD2 (Xu et al., 2024), miR-192–5p targeted interleukin-17 receptor A (Li et al., 2021), and engineered delivery of miR-141–3p (Meng et al., 2024) or miR-29a (Yuan et al., 2021) reduced TGF-β2/SMAD-dependent activation in hypertrophic-scar fibroblasts. Screening of EVs from amniotic-fluid stem cells identified additional miRNAs that limited TGF-β1-induced myofibroblast conversion (Zhang et al., 2021b). These studies converge on the same signaling network, although the contribution of each miRNA to unmodified EV preparations is likely to vary.

Donor condition can reverse the direction of the response. EVs from diabetic adipose-derived stem cells increased TGF-β1 secretion by resident monocytes or macrophages and enhanced TGF-β/SMAD3-dependent fibroblast activation during diabetic repair (Hsu et al., 2022). This result contrasts with the antifibrotic activity of healthy or engineered ADSC-EVs and shows that a shared source label does not define a uniform product. It also places macrophages between the EV source and the fibroblast response, highlighting an indirect route to fibroblast activation.

Outside the TGF-β axis, scar studies converge on PI3K/AKT/mTOR- and NF-κB-related signaling. ADSC-EVs reduced PI3K/AKT/mTOR-associated inflammation, mitochondrial dysfunction, and collagen accumulation in keloid fibroblasts (Liu C. et al., 2024). Other MSC-EV preparations inhibited hypertrophic-scar fibroblast proliferation through the tumor necrosis factor superfamily member 13/heparan sulfate proteoglycan 2 pathway (Zhang et al., 2023), whereas miR-138–5p and miR-181a were each linked to sirtuin 1 (SIRT1)-dependent attenuation of profibrotic fibroblast activity and scar formation (Zhao et al., 2022; Chen et al., 2023). Despite this pathway-level convergence, the studies differ in rescue design, EV dose, cell model, and outcome measure. The evidence therefore favors a set of recurrent signaling modules rather than a universal hierarchy of cargo molecules.

Mechanical signaling adds another layer because matrix stiffening can maintain fibroblast activation after soluble profibrotic stimuli decline. Integrin–Rho-associated kinase, focal-adhesion, and Yes-associated protein/transcriptional coactivator with PDZ-binding motif (YAP/TAZ) pathways translate stiffness into contractile and matrix-producing transcriptional programs (Di et al., 2023; Burridge et al., 2019). Adipose-tissue EV-associated miR-92a promoted repair through large tumor suppressor kinase 2 (LATS2)/Hippo-YAP signaling (Shao et al., 2024), but whether EV treatment can durably reverse established mechanical feedback in mature scars remains unresolved.

### 3.3 EV regulation of fibroblast survival, death, and senescence

#### 3.3.1 Evidence in wound healing

Fibroblast survival is often beneficial in diabetic, irradiated, and aged wounds, where oxidative stress and premature cell loss limit closure. ADSC-EVs restored fibroblast viability under high-glucose conditions (Yang et al., 2024; Tian et al., 2025) and reduced apoptosis- and pyroptosis-associated proteins after irradiation (Liu et al., 2025b). Platelet-rich plasma-derived EVs delivered in a hydrogel suppressed fibroblast ferroptosis and improved diabetic wound healing (Wang S. et al., 2025). Because the hydrogel itself can alter EV retention and the local inflammatory environment, carrier and EV effects should be evaluated separately.

EVs can also modify age-related dysfunction. EVs from young fibroblasts increased migration and myofibroblast conversion in aged wounds (Xia et al., 2022), whereas EVs from three-dimensional cultured dedifferentiated fat cells reduced reactive oxygen species and senescence markers while improving repair (Ma et al., 2025). These interventions may correct an inadequate early reparative response, but the accompanying increase in myofibroblast conversion makes later remodeling an important endpoint.

#### 3.3.2 Evidence in scar formation

During late remodeling, prolonged survival of activated fibroblasts may become detrimental. Resolution of granulation tissue requires apoptosis or deactivation of excess myofibroblasts (Desmoulière et al., 1995), whereas apoptosis resistance and senescence-associated secretory programs can sustain fibrosis and inflammation (Gorgoulis et al., 2019; Lin and Xu, 2020). Current scar studies rarely test whether EVs directly promote myofibroblast clearance. Short-term reductions in α-smooth muscle actin or collagen therefore provide limited information about mature matrix architecture, tissue mechanics, recurrence, or durable resolution.

**FIGURE 2:**
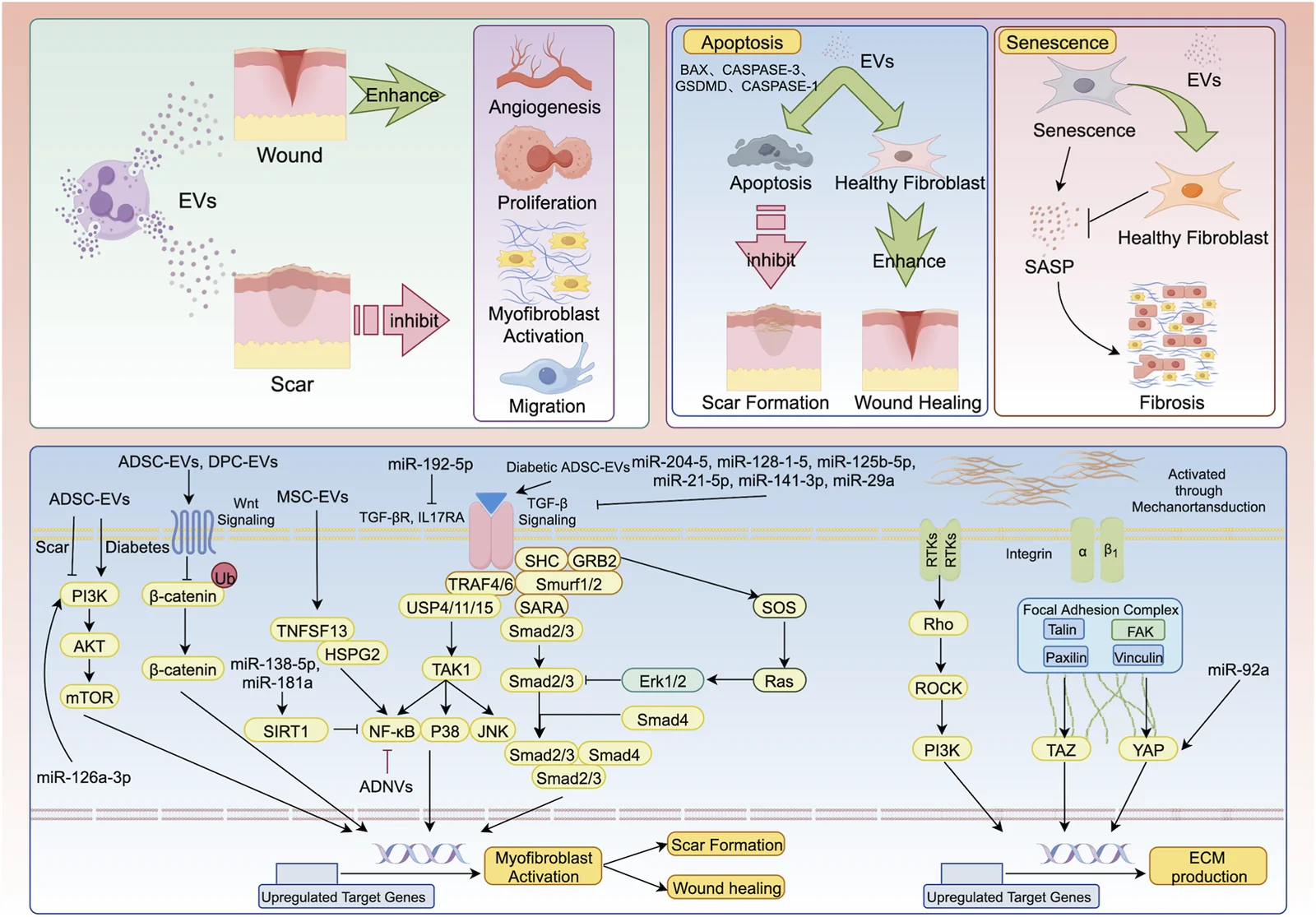
EVs regulate fibroblast proliferation, activation, fate transitions, and matrix remodeling during wound healing and scar formation. During wound healing and scar formation, extracellular vesicles derived from different sources and their cargos act on fibroblasts and modulate their functions and fate through multiple signaling pathways, thereby determining whether they promote wound repair or restrain continued scar overgrowth. Abbreviations: ADNVs, apple-derived nanovesicles; ADSC-EVs, extracellular vesicles from adipose-derived stem cells; AKT, protein kinase B; BAX, BCL2-associated X protein; DPC-EVs, extracellular vesicles from dermal papilla cells; ECM, extracellular matrix; ERK, extracellular signal-regulated kinase; FAK, focal adhesion kinase; GRB2, growth factor receptor-bound protein 2; GSDMD, gasdermin D; HSPG2, heparan sulfate proteoglycan 2; mTOR, mechanistic target of rapamycin; NF-κB, nuclear factor kappa B; PI3K, phosphoinositide 3-kinase; ROCK, Rho-associated kinase; RTK, receptor tyrosine kinase; SARA, SMAD anchor for receptor activation; SASP, senescence-associated secretory phenotype; SHC, Src homology 2 domain-containing transforming protein; SIRT1, sirtuin 1; SMAD, SMAD family proteins; SMURF, SMAD ubiquitination regulatory factor; SOS, son of sevenless; TAK1, transforming growth factor-beta-activated kinase 1; TAZ, transcriptional coactivator with PDZ-binding motif; TGF-βR, transforming growth factor-beta receptor; TNFSF13, tumor necrosis factor superfamily member 13; TRAF, tumor necrosis factor receptor-associated factor; Ub, ubiquitin; USP, ubiquitin-specific peptidase; YAP, Yes-associated protein. Created with Figdraw.

Overall, EVs regulate fibroblast proliferation, activation, apoptosis, senescence, and matrix remodeling through diverse signaling pathways, thereby influencing the balance between effective repair and fibrotic scar formation (Figure 2).

## 4 EV-mediated crosstalk between macrophages and fibroblasts in wound healing and scar formation

### 4.1 Crosstalk between macrophages and fibroblasts in wound healing and scar formation

**FIGURE 3:**
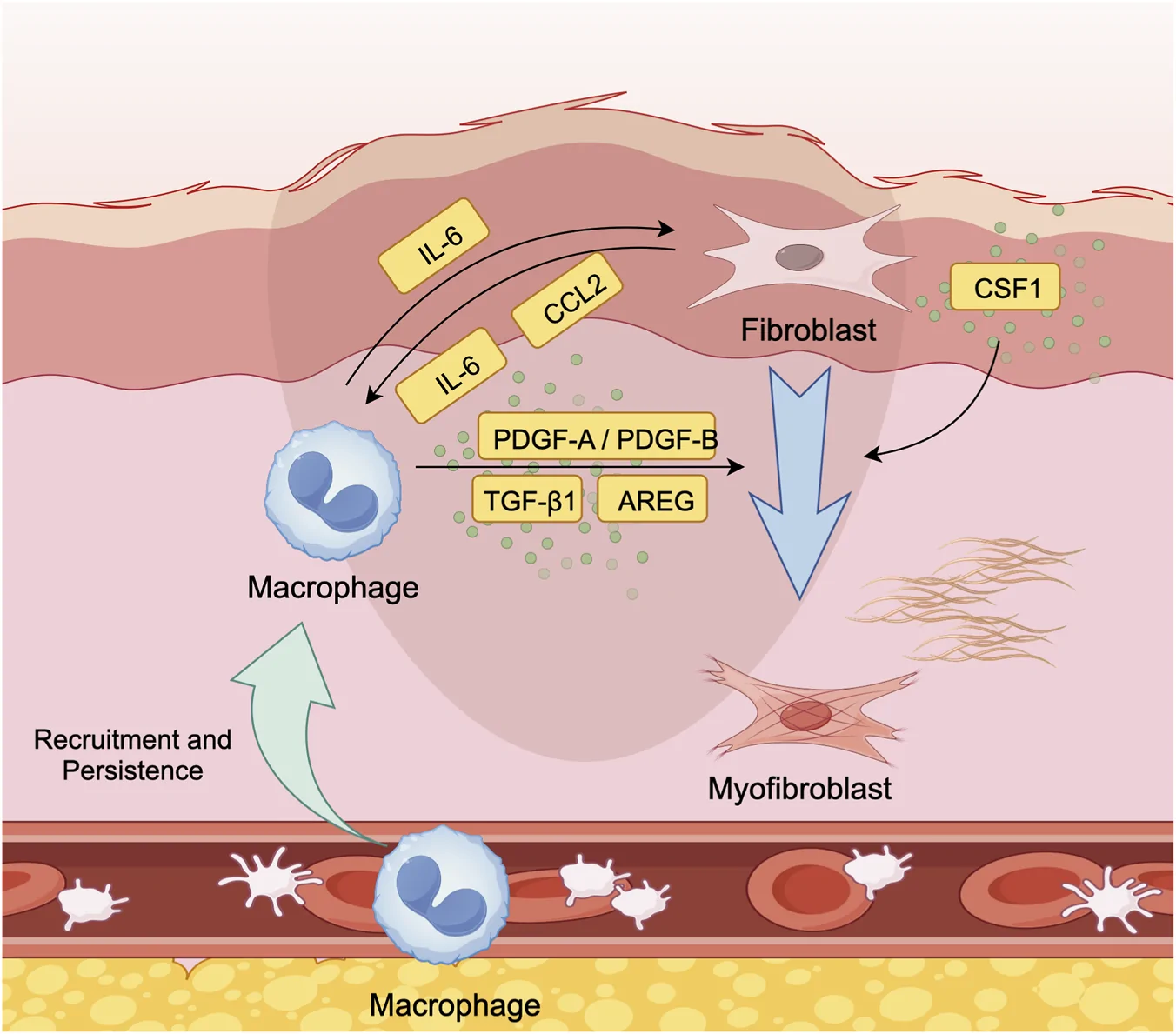
Fibroblast–macrophage interactions in wound healing and scar formation. Fibroblasts support macrophage recruitment through CCL2 and maintenance through CSF1, and both cell types participate in IL-6-mediated signaling. During skin repair, macrophages support fibroblast proliferation and heterogeneity through PDGF-associated signaling. Macrophage-derived TGF-β1 can activate fibroblasts in non-cutaneous injury models, and AREG is shown in the macrophage-to-stromal direction. Abbreviations: AREG, amphiregulin; CCL2, C-C motif chemokine ligand 2; CSF1, colony-stimulating factor 1; ECM, extracellular matrix; IL-6, interleukin-6; PDGF, platelet-derived growth factor; TGF-β1, transforming growth factor-beta 1. Created with Figdraw.

Reciprocal macrophage–fibroblast signaling is well established in skin even when EV involvement has not been demonstrated. During skin repair, macrophages support myofibroblast proliferation and heterogeneity through PDGF-associated signaling (Shook et al., 2018). Conversely, conditional loss of fibroblast-derived colony-stimulating factor 1 (CSF1) reduces CD64^+^ and CD11c^+^ macrophages and impairs wound healing (Vollmers et al., 2026), while fibroblast-derived C-C motif chemokine ligand 2 (CCL2) supports monocyte and macrophage recruitment during acute repair (Amuso et al., 2025). Macrophage-derived interleukin-6 (IL-6) can restore selected matrix-remodeling features of healing-associated fibroblasts in diabetic wounds (Xiao et al., 2024). These observations define a cutaneous cellular circuit within which EV-mediated mechanisms may operate, but they should not be interpreted as evidence of EV transfer (Figure 3).

Related non-cutaneous studies identify additional candidate signals. Macrophage-derived TGF-β1 directly activates fibroblasts after ureteral injury (Ueshima et al., 2019), and macrophage-derived amphiregulin activates latent TGF-β through integrin αV in pericytes (Minutti et al., 2019). In cardiac fibrosis, macrophage stimulation induces fibroblast IL-6 that is required for TGF-β/SMAD activation (Ma et al., 2012), while inflammatory synovial fibroblasts can reprogram macrophage glycolysis, mitochondrial respiration, and nutrient uptake (Saeki and Imai, 2020). These pathways provide mechanistic comparators for skin but require cutaneous validation before they are incorporated into EV-specific models.

Spatially resolved and perturbational studies further define the cutaneous settings in which macrophage–fibroblast interactions occur. In diabetic foot ulcers, healing-associated fibroblasts were concentrated in the wound bed, while macrophage-state composition differed between healing and non-healing ulcers (Theocharidis et al., 2022). Complementary functional evidence in normal and injured skin comes from fibroblast-restricted CSF1 deletion, which reduced CD64^+^ and subsequently CD11c^+^ macrophages, delayed repair, and altered fibroblast cell-cycle, metabolic, ECM, and immune programs (Vollmers et al., 2026). More recently, multiplex imaging and spatial transcriptomics identified an early nerve-associated repair-glia niche that recruits monocytes/macrophages before broader stromal expansion, further illustrating the multicellular and spatially ordered nature of early repair (Stierli et al., 2026).

Fibrotic human skin provides additional spatial support for macrophage–fibroblast coupling outside the wound setting. In diffuse cutaneous systemic sclerosis, spatial multiomics identified expanding fibrotic niches co-enriched for fibroblasts and macrophages whose abundance correlated with clinical severity; atypical chemokine receptor 3 (ACKR3) expression in myofibroblast progenitors was linked to C-X-C motif chemokine ligand 12 (CXCL12)/C-X-C chemokine receptor 4 (CXCR4)-dependent recruitment of proinflammatory macrophages, and CXCR4 inhibition attenuated experimental skin and lung fibrosis (Li Z. et al., 2025). An independent study of early, treatment-naive diffuse cutaneous systemic sclerosis identified a proinflammatory vascular niche in which PDGF-expressing monocyte-derived mannose receptor C-type 1 (MRC1)+macrophages localized near PDGFR^hi^THY1^hi^ fibroblasts, with fibroblast, macrophage, and T-cell colocalization around the vasculature (Jarnagin et al., 2026). These spatial relationships identify candidate cellular contexts for reciprocal signaling in fibrotic skin but do not themselves establish EV transfer.

Macrophage-to-myofibroblast transition (MMT) has been proposed as an additional source of myofibroblasts, mainly in renal, pulmonary, and other solid-organ models (Yang et al., 2021; Vierhout et al., 2021; Xiong et al., 2021; Torres et al., 2020; Cheng et al., 2021). Co-expression of macrophage and myofibroblast markers can also arise from phagocytosis, cell fusion, or transient marker expression and is not sufficient to establish lineage conversion. Single-cell profiles of diabetic foot ulcers reveal heterogeneous immune and stromal states but provide no lineage evidence for MMT (Theocharidis et al., 2022). In skin, MMT should therefore be regarded as a candidate mechanism until direct lineage-tracing studies demonstrate macrophage-derived myofibroblasts in cutaneous repair or scarring.

### 4.2 Macrophage-derived EVs regulate fibroblasts

Rather than sharing a single profibrotic cargo, macrophage EVs from different models carry distinct signals that converge on fibroblast metabolism, survival pathways, autophagy, and ECM production. The available studies also differ in how directly they establish EV-dependent donor-to-recipient signaling.

In a TGF-β1-stimulated coculture system, EVs from M2-like macrophages transferred the long noncoding RNA ASLNCS5088 to dermal fibroblasts. ASLNCS5088 relieved miR-200c-3p-mediated repression of glutaminase, increased α-smooth muscle actin and collagen expression, and promoted fibroblast proliferation and invasion; inhibition of EV generation with GW4869 attenuated these effects *in vitro* and reduced late scar fibrosis *in vivo* (Chen et al., 2019). A separate hypertrophic-scar study identified EV-associated LINC01605 as a regulator of the miR-493–3p/AKT serine/threonine kinase 1 (AKT1) axis. LINC01605 transfer increased AKT/mTOR signaling, fibroblast proliferation, migration, invasion, and type I and III collagen expression (Zhu et al., 2021). Together, the two studies link different long noncoding RNA cargos to metabolic and growth-signaling programs in scar fibroblasts.

The microenvironment further modifies EV activity. Hypoxic macrophage EVs were enriched in miR-26b-5p, which targeted phosphatase and tensin homolog (PTEN) and activated PI3K/AKT signaling in keloid fibroblasts, increasing proliferation, migration, and invasion (Dai et al., 2024). In hypertrophic-scar fibroblasts, CXCL2-containing macrophage EVs activated the C-X-C chemokine receptor 7/PI3K/mTOR axis, increased autophagy, and promoted collagen deposition; pharmacological inhibition of autophagy reduced the response (Shi et al., 2024). Both pathways converge on PI3K/AKT/mTOR-related signaling despite differences in cargo and conditioning stimulus. These data show that hypoxia and TGF-β-rich conditions alter selected EV cargos and downstream fibroblast responses. An M2-like label is therefore too broad to serve as a mechanistic designation.

Interpretation of these scar studies is shaped by several design features. Macrophage states were generated with operational M1/M2 protocols, recipient cells were usually bulk-cultured fibroblasts, and major endpoints included proliferation, migration, collagen expression, and α-smooth muscle actin. GW4869 supported a role for vesicle release in the ASLNCS5088 and LINC01605 studies but did not resolve which EV subtype mediated the response (Chen et al., 2019; Zhu et al., 2021). Some studies included *in vivo* scar models, yet effects on mature scar architecture, stiffness, and recurrence in humans remain unknown.

Other reports describe parallel effects on macrophages and fibroblasts without tracing direct transfer between them. A composite system combining M2-like macrophage EVs with two-dimensional transition-metal carbides increased angiogenesis, fibroblast migration, and repair-associated macrophage features in diabetic wounds (Jiang et al., 2024). An acellular dermal matrix functionalized with EVs from M2-like macrophages enhanced the proliferation and migration of human dermal fibroblasts and keratinocytes and showed immunoregulatory and angio-inductive activity in a skin tissue-engineering model (Yazicioglu et al., 2026). These biomaterial constructs are relevant to therapeutic delivery; however, because the carrier itself alters EV retention and the local microenvironment, the observed benefit primarily supports the therapeutic value of the composite delivery system rather than physiological macrophage-to-fibroblast EV communication.

### 4.3 Fibroblast-derived EVs regulate macrophages

Sequential studies of fibroblast-to-macrophage signaling show that EV activity changes across the repair process. Dermal fibroblasts promoted repair-associated macrophage activation in impaired wounds through both EV and non-EV mechanisms (Ferrer et al., 2017). A subsequent study isolated the EV contribution and found that fibroblast-secreted EVs enhanced inflammatory macrophage responses early after injury and favored repair-associated responses later in healing. Inhibiting EV release disrupted this temporal coordination, whereas EV supplementation improved macrophage-state switching and wound repair in diabetic models (Chen et al., 2025). The findings suggest that fibroblast EVs amplify phase-appropriate local cues rather than impose a fixed M1-like or M2-like state.

Disease-associated fibroblasts can produce a different response. In diabetic foot ulcers, fibroblast EV-associated miR-93–5p targeted autophagy-related 16-like 1 (ATG16L1) in macrophages, impaired autophagic clearance of reactive oxygen species, increased NLR family pyrin domain containing 3 (NLRP3) inflammasome activity, and delayed healing. Antagonizing miR-93–5p restored macrophage autophagy and improved repair (Xu et al., 2025). Target validation and antagonist rescue support a causal role for pathological fibroblast EVs in maintaining macrophage inflammatory activity.

Fibroblast EVs may also initiate a secondary feedback loop in skin fibrosis. EVs from systemic sclerosis dermal fibroblasts were taken up by macrophages and induced a mixed activation program with increased inflammatory and profibrotic mediator release. Signals from these EV-activated macrophages subsequently increased collagen and fibronectin expression in fibroblasts, whereas direct exposure of fibroblasts to the same EV preparation produced a weaker response (Bhandari et al., 2023). Although systemic sclerosis differs from wound and hypertrophic-scar models, this human skin study describes a feedback circuit in which fibroblast-derived EVs activate macrophages, which subsequently enhance profibrotic fibroblast responses.

Fibroblast-derived EVs therefore have state-dependent immunological effects. EVs from fibroblasts engaged in orderly repair can reinforce phase-appropriate macrophage responses, whereas EVs from diabetic ulcers or fibrotic skin can impair autophagy and sustain inflammatory–fibrotic feedback. The state of the producing fibroblast is likely to be a key determinant of the macrophage response, but direct comparisons across fibroblast states are still needed.

### 4.4 Time-dependent, indirect, and cross-tissue evidence

Cross-tissue studies provide additional candidate mechanisms for skin. In endometrial fibrosis, tumor necrosis factor-stimulated gene 6-modified EV therapy required macrophage regulation to suppress stromal fibroblast activation (Sun et al., 2024). In osteoarthritis, inflammatory fibroblast-like synoviocyte EVs increased macrophage glycolysis and inflammatory polarization (Liu B. et al., 2024), while macrophage EV transfer of angiotensin II type 1 receptor generated a positive feedback loop with fibroblasts in bleomycin-induced pulmonary fibrosis (Sun et al., 2021). These models illustrate macrophage-dependent antifibrotic and stromal–immunometabolic mechanisms; whether equivalent circuits operate in wounds or pathological scars remains to be tested.

Direct time-course evidence remains scarce in cutaneous EV crosstalk studies. The fibroblast-EV study described above compared early and late wound phases and demonstrated a corresponding shift in macrophage responses (Chen et al., 2025), whereas most other reports compare different EV sources or disease models. Whether a given EV axis changes from reparative to fibrogenic as healing progresses therefore remains largely untested within the same experimental system.

**FIGURE 4:**
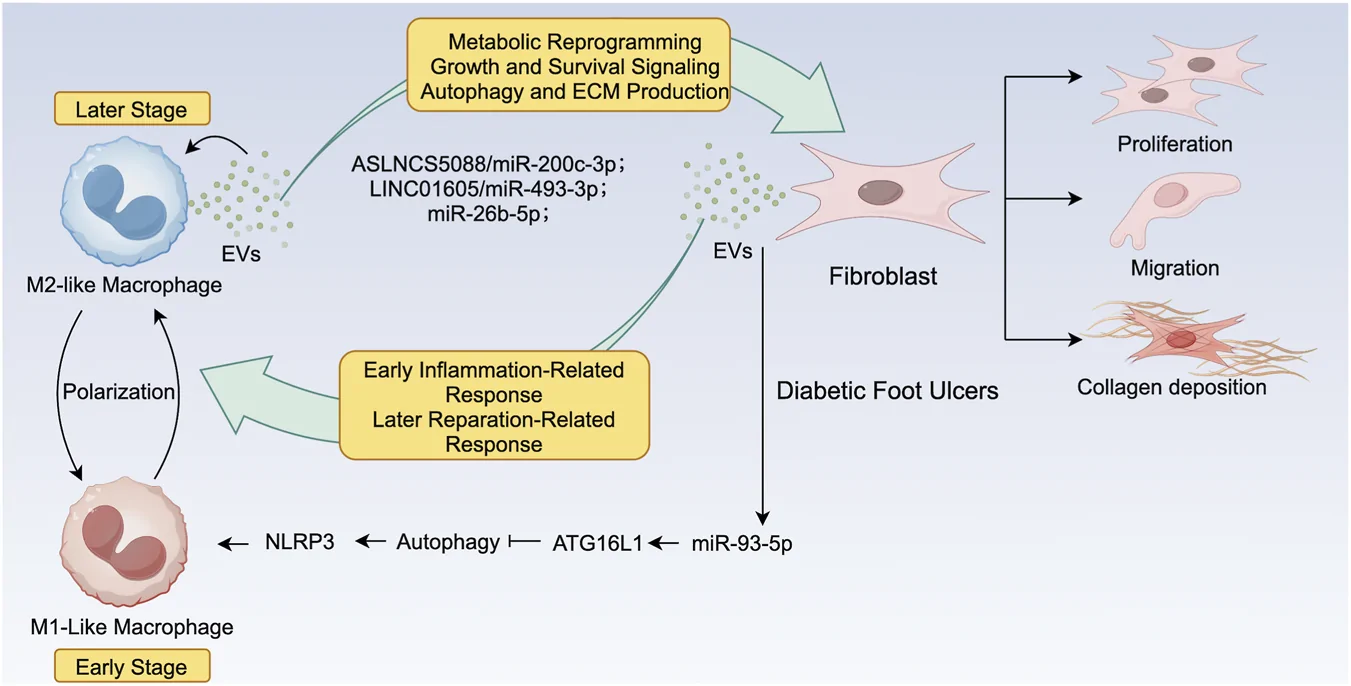
EV-mediated crosstalk between macrophages and fibroblasts in wound healing and scar formation. Fibroblast-derived EVs amplify inflammatory-associated macrophage responses during early repair and repair-associated responses later in healing. In pathological fibroblast EVs, miR-93–5p suppresses autophagy-related 16-like 1 (ATG16L1), impairing autophagy-dependent reactive oxygen species (ROS) clearance and enhancing NLR family pyrin domain containing 3 (NLRP3) inflammasome activation. In the reciprocal direction, representative macrophage-derived EV axes—including ASLNCS5088/miR-200c-3p/glutaminase, LINC01605/miR-493–3p/AKT1, miR-26b-5p/PTEN/PI3K/AKT, and CXCL2/CXCR7/mTOR—converge on fibroblast metabolism, growth and survival signaling, autophagy, and extracellular matrix production. Abbreviations: AKT, protein kinase B; AKT1, AKT serine/threonine kinase 1; ATG16L1, autophagy-related 16-like 1; CXCL2, C-X-C motif chemokine ligand 2; CXCR7, C-X-C chemokine receptor type 7; ECM, extracellular matrix; mTOR, mechanistic target of rapamycin; NLRP3, NLR family pyrin domain containing 3; PI3K, phosphoinositide 3-kinase; PTEN, phosphatase and tensin homolog; ROS, reactive oxygen species. Created with Figdraw.

Current evidence supports EV-mediated communication in both macrophage-to-fibroblast and fibroblast-to-macrophage directions, but the strength of support varies substantially across studies. Table 1 summarizes representative cutaneous studies according to the extent to which EV-dependent donor–recipient communication was experimentally demonstrated (Figure 4).

**TABLE 1:** Strength of evidence for representative EV-mediated macrophage–fibroblast communication studies in cutaneous wound healing and scarring.

Direction	EV source/model	Recipient	Representative mechanism	Causal feature	Evidence category
Macrophage → fibroblast	M2-like macrophage EVs; TGF-β1-stimulated coculture (Chen et al., 2019)	Dermal fibroblast	ASLNCS5088/miR-200c-3p/glutaminase	GW4869 blockade of EV generation attenuated recipient response	Direct
Macrophage → fibroblast	M2-like macrophage EVs; hypertrophic scar (Zhu et al., 2021)	Dermal fibroblast	LINC01605/miR-493–3p/AKT1	GW4869 blockade impaired macrophage-induced fibroblast phenotype	Direct
Macrophage → fibroblast	Hypoxic macrophage EVs; keloid (Dai et al., 2024)	Keloid fibroblast	miR-26b-5p/PTEN/PI3K-AKT	Purified EV exposure with cargo/target validation; no endogenous transfer blockade	Supportive
Macrophage → fibroblast	M2-like macrophage EVs; hypertrophic scar (Shi et al., 2024)	Hypertrophic-scar fibroblast	CXCL2/CXCR7/mTOR/autophagy	Purified EV exposure and pathway perturbation; no endogenous transfer blockade	Supportive
Macrophage → fibroblast	MXene–M2-like macrophage EV composite; diabetic wound (Jiang et al., 2024)	Multiple wound cell types	PI3K/AKT-associated repair	Biomaterial composite; no donor-to-recipient EV transfer demonstrated	Correlative/composite
Fibroblast → macrophage	Dermal fibroblast EVs; diabetic wound time course (Chen et al., 2025)	Macrophage	Phase-dependent inflammatory-to-repair response	EV-release inhibition and EV supplementation altered macrophage-state dynamics	Direct
Fibroblast → macrophage	Diabetic foot ulcer fibroblast EVs (Xu et al., 2025)	Macrophage	miR-93–5p/ATG16L1/autophagy/NLRP3	Cargo antagonism/rescue supports EV-cargo mechanism; endogenous transfer dependence less directly tested	Supportive
Fibroblast → macrophage	Systemic sclerosis fibroblast EVs (Bhandari et al., 2023)	Macrophage, then fibroblast	Inflammatory/profibrotic feedback	EV uptake and secondary conditioned-media response; no EV-release blockade	Supportive
Macrophage → fibroblast	M2-like macrophage EV-functionalized acellular dermal matrix (Yazicioglu et al., 2026)	Fibroblast/keratinocyte	Repair and angiogenic responses	Carrier-EV composite; no physiological donor-to-recipient transfer demonstrated	Correlative/composite

Evidence category refers specifically to support for EV-dependent intercellular communication, not to overall study quality. Direct evidence requires a defined donor–recipient relationship together with functional perturbation of EV release or uptake (or an equivalent dependency test); supportive evidence demonstrates activity of an isolated EV preparation in a defined recipient without establishing that endogenous donor-to-recipient transfer is required; correlative/composite evidence describes parallel cellular effects or EV–biomaterial constructs without demonstrated intercellular EV dependence. Cargo depletion or rescue strengthens mechanistic attribution but is considered separately from evidence of endogenous EV transfer.

## 5 Discussion

### 5.1 Integrated mechanistic interpretation

The diverse cargos described above converge on a limited set of interacting regulatory modules. NF-κB, JAK/STAT, autophagy, inflammasome, and redox pathways shape macrophage inflammatory activity and stress adaptation. PI3K/AKT/mTOR and extracellular signal-regulated kinase signaling recur in macrophage autophagy and in fibroblast migration, survival, and fibrotic activity. TGF-β/SMAD signaling governs myofibroblast differentiation and matrix production, whereas Wnt/β-catenin, integrin–Rho-associated kinase, and Hippo-YAP pathways connect regenerative signaling and matrix mechanics to fibroblast behavior. Organizing the evidence around these modules clarifies common mechanisms across otherwise diverse cargo studies.

These modules interact within the wound environment. Macrophage-derived TGF-β1, PDGF-associated skin-repair signals, and amphiregulin influence fibroblast activation and growth-factor sensitivity. Matrix stiffening reinforces focal-adhesion, Rho-associated kinase, and YAP/TAZ signaling in fibroblasts. Activated fibroblasts in turn release CSF1, CCL2, IL-6, EVs, and metabolic cues that alter macrophage recruitment and function. The same signaling module may have different consequences across repair stages: PI3K/AKT activity can restore migration in a diabetic wound, whereas persistent PI3K/AKT/mTOR signaling is associated with keloid fibroblast activity. Similarly, TGF-β supports matrix restoration but becomes profibrotic when excessive or prolonged. Because most studies examine one phase or disease context, the stage-dependent model remains a synthesis rather than a directly tested sequence.

Although non-coding RNAs dominate many mechanistic reports, biologically active non-RNA components are also evident. Surface-associated HSP90 on ADSC-EVs activates LRP1/AKT signaling in diabetic wounds (Ren et al., 2022), whereas CXCL2-containing macrophage EVs engage CXCR7/mTOR signaling in hypertrophic-scar fibroblasts (Shi et al., 2024). HSP72/phosphatidylinositol transfer protein and semaphorin 7A provide additional protein-associated examples (Kim et al., 2021) (Cho et al., 2025). EV activity can also depend on molecules associated with the vesicle surface: removal of a functional protein corona from stromal-cell EV preparations reduced their angiogenic activity in a skin-regeneration model (Wolf et al., 2022). Defined lipid mechanisms remain poorly resolved in cutaneous macrophage–fibroblast communication. In a non-cutaneous regenerative model, macrophage EV-associated cholesterol was required for biological activity, indicating that EV lipid composition can contribute directly to function (Vanherle et al., 2023); comparable causal evidence for defined lipid species in wound or scar macrophage–fibroblast models is currently lacking. Defined EV-associated metabolites have been studied even less extensively in cutaneous macrophage–fibroblast communication, and their functional contribution remains largely unresolved. Together, these observations support a multicomponent model of EV activity in which surface-associated proteins, luminal proteins, RNAs, membrane lipids, and metabolites may act in combination. Accordingly, enrichment of an individual miRNA should not be equated with functional dominance unless its necessity is demonstrated by depletion or rescue experiments.

Divergent EV outcomes can often be traced to the state of the producing cell, EV composition and dose, the phenotype and matrix environment of the recipient cell, and the timing of exposure. The opposite effects of healthy and diabetic ADSC-EVs on TGF-β-associated repair illustrate how donor condition can outweigh a shared source label (Hsu et al., 2022). This context dependence should be considered when comparing EV preparations across acute wounds, diabetic ulcers, hypertrophic scars, and keloids.

Causal evidence for reciprocal EV communication remains uneven. Demonstrating that an isolated EV preparation alters a recipient cell establishes EV bioactivity but does not establish that endogenous donor-to-recipient EV transfer is required in tissue. Stronger designs combine cell-specific labeling or uptake measurements with selective interference in EV release or uptake and, where a specific cargo is implicated, depletion and rescue within the same donor–recipient system. Only a subset of cutaneous macrophage–fibroblast studies currently incorporate multiple elements of this causal chain. Parallel changes in macrophage and fibroblast endpoints, and particularly EV–biomaterial composite studies, should therefore be interpreted as therapeutic or correlative evidence unless intercellular EV dependence is directly tested.

### 5.2 Translational opportunities and barriers

Clinical EV studies have demonstrated analytical feasibility for early lung-cancer detection (Han et al., 2025), monitoring oligorecurrent prostate cancer (Andrews et al., 2025), and predicting immune effector cell-associated neurotoxicity syndrome after chimeric antigen receptor T-cell therapy (Storci et al., 2024). Therapeutic EV or microparticle products have also entered clinical evaluation for malignant pleural effusion (Zeng et al., 2026), inflammatory disease (Hyun et al., 2025), osteoarthritis (Wang Y. et al., 2025), and pulmonary fibrosis (Li M. et al., 2025). These studies establish clinical feasibility in other indications, but their relevance to wound healing and scar prevention remains indirect.

Clinical evidence in skin remains preliminary. A first-in-human study of allogeneic platelet-derived EVs found cutaneous application feasible and tolerable in healthy participants but did not test accelerated healing in patients (Johnson et al., 2023). A randomized study of Wharton’s jelly mesenchymal stromal cell EVs in diabetic foot ulcers reported improved closure (Kishta et al., 2025), although independent replication, product comparability, and longer follow-up are still needed. A topical MSC-EV ointment study in psoriasis primarily informs short-term safety rather than efficacy in wounds or scars (Chandran et al., 2025). Published clinical studies directly relevant to cutaneous EV administration are summarized in Table 2.

**TABLE 2:** Published clinical studies directly relevant to cutaneous EV administration.

EV source	Indication	Design/phase	N	Primary endpoint	Key findings	Follow-up
Allogeneic platelet EVs (Johnson et al., 2023)	Healthy volunteers; paired 4-mm punch wounds	Randomized double-blind placebo-controlled phase I	11	Safety and tolerability	No serious safety signal; mean healing time 22.8 ± 8.7 days in both EV and placebo wounds	All wounds closed by day 30
Wharton’s jelly MSC-derived exosomes (Kishta et al., 2025)	Persistent diabetic foot ulcers	Randomized double-blind controlled trial	110	Percentage reduction in ulcer area at 16 weeks	Higher complete-healing proportion; mean time to complete healing 6 weeks versus 20 weeks in controls	Serial evaluations reported through 6 months
MSC exosome ointment (Chandran et al., 2025)	Healthy volunteers; topical product developed for psoriasis	Single-center open-label phase I	10	Safety and tolerability	Topical administration was tolerated; efficacy was not tested in this phase I study	20-day treatment period

EV heterogeneity is a major translational challenge. Donor age, disease, metabolic state, tissue source, cell passage, culture medium, oxygen tension, and preconditioning can alter EV yield and composition (Cook and Li, 2025; Ebr et al., 2024). The contrasting effects of healthy and diabetic ADSC-EVs on TGF-β-associated repair show that cellular source alone is insufficient to define an EV product. Clinical development therefore requires qualified donor cells, controlled culture conditions, mechanism-linked potency assays, clear product classification, good manufacturing practice-compliant production, comparability assessment after manufacturing changes, and prespecified release specifications (Welsh et al., 2024; Ebr et al., 2024).

Three-dimensional culture can alter donor-cell phenotype, EV biogenesis, and product potency. EVs from three-dimensional cultured dedifferentiated fat cells reduced senescence-associated dysfunction in aged wounds (Ma et al., 2025). A self-feeder layer three-dimensional (SFL-3D) system was subsequently applied to dermal papilla cells, and the resulting three-dimensional dermal papilla cell-derived EVs (tdDPC-EVs) showed stronger proangiogenic activity than conventional dermal papilla cell-derived EVs (DPC-EVs) and improved full-thickness wound repair through Krüppel-like factor 4 (KLF4)/vascular endothelial growth factor A (VEGFA)-associated signaling (Wang et al., 2023b). More recent SFL-3D studies have extended this approach to scar and diabetic-wound models. In hypertrophic-scar fibroblasts, SFL-3D supported prolonged dermal papilla-cell expansion and enhanced EV biogenesis; tdDPC-EVs enriched in miR-26a-5p suppressed proliferation, migration, myofibroblast differentiation, and collagen production through the cyclin E2 (CCNE2)/PI3K/AKT axis, with gain- and loss-of-function experiments supporting this cargo–target relationship and a rabbit-ear model confirming antiscarring activity (Wang et al., 2026a). SFL-3D has also been applied to adipose-derived mesenchymal cells, whose EVs reduced high-glucose-induced dermal microvascular endothelial senescence, oxidative stress, and mitochondrial dysfunction through PI3K/AKT/mTOR/eukaryotic translation initiation factor 4E-binding protein 1 (4EBP1) signaling and outperformed conventional ADSC-EVs in diabetic mouse and Bama miniature-pig wound models, including measures of wound closure, neovascularization, scar width, and collagen organization (Wang et al., 2026b). These studies support culture architecture as a controllable determinant of EV potency, while current evidence remains insufficient to establish SFL-3D as a general manufacturing standard.

Isolation and characterization must balance purity, recovery, and scale. Ultracentrifugation, precipitation, size-exclusion chromatography, affinity capture, and combined methods recover different EV fractions and co-isolate different non-vesicular components (Reiner et al., 2017). MISEV2023 therefore recommends transparent reporting of source material, separation procedures, particle and protein measurements, EV-associated markers, contaminants, storage, and functional testing (Welsh et al., 2024). Product-specific acceptance ranges and reference standards will still need to be defined for clinical development.

Dose metrics are not interchangeable. Particle number, total protein, source-cell equivalents, and administered volume describe different product properties, and none directly measures delivery of an active cargo (Nguyen et al., 2020). Potency assays should therefore reflect the intended clinical action, such as resolving inflammation, restoring fibroblast migration in a chronic wound, or suppressing persistent myofibroblast activation in a scar (Lener et al., 2015). Without a mechanism-linked assay, nominally equivalent doses may not have equivalent biological activity.

Local hydrogels, microneedles, ointments, and dressings may improve retention and reduce systemic exposure. Hydrogels can increase local residence and modify release kinetics, whereas microneedles and dressings can alter penetration depth and spatial distribution. Delivery systems can also change local exposure, degradation, inflammation, and the contribution of biomaterial components (Meng et al., 2024; Wang S. et al., 2025; Jiang et al., 2024; Chandran et al., 2025). EV–carrier studies therefore require matched carrier-only controls and reporting of release kinetics, EV integrity after formulation, local retention, and recipient-cell uptake. Therapeutic benefit from an EV–biomaterial composite should not be attributed to the EV preparation alone unless the carrier contribution is separated experimentally. Safety and product-quality assessment should cover off-target biodistribution, excessive angiogenesis, immunomodulation, transmission of pathological donor signals, unintended fibrosis, storage and freeze-thaw stability, transport, and compatibility with the delivery material.

### 5.3 Priorities for future research

Recent reviews have largely organized cutaneous EV biology by EV source, wound-healing phase, therapeutic platform, or scar-associated cell type (Eerdekens et al., 2025) (Shi et al., 2025) (Sadeghi et al., 2025) (Lu et al., 2026). A reciprocal macrophage–fibroblast perspective provides a complementary framework because both donor and recipient states evolve during repair, allowing EV-associated signals to be interpreted within the changing immune–stromal context of wound healing and scar formation.

Future studies should test macrophage–fibroblast EV exchange directly in skin. Cell-type-specific EV labeling, conditional inhibition of EV release, uptake blockade, cargo depletion, and rescue are most informative when combined within the same model. Cross-tissue mechanisms can guide candidate selection, but cutaneous validation requires identification of the producing cell, recipient cell, and transferred cargo.

Study design should also capture cellular heterogeneity and time. Single-cell and spatial studies have identified wound-associated fibroblast and immune states that are obscured in bulk cultures. Spatial mapping and serial sampling can determine which states produce or receive EVs and separate early inflammatory effects from later remodeling or recurrence. Comparisons across acute wounds, diabetic ulcers, hypertrophic scars, and keloids would benefit from matched sampling windows and functional endpoints.

Comparative studies will be most informative when reporting practices, dose metrics, and functional endpoints are harmonized. Cargo–target relationships should be prioritized when supported by target engagement, necessity, rescue, and replication across independent EV preparations. Additional expression-based cargo lists are unlikely to explain why nominally similar EV sources produce different outcomes without corresponding functional validation.

## 6 Conclusion

EVs regulate macrophages, fibroblasts, and their interactions during wound healing and pathological scarring, with effects that vary by cellular context and repair phase. Direct regulation of each cell population is supported by a broader evidence base than bidirectional EV crosstalk in skin. Progress toward clinical use will depend on causal validation, reproducible product characterization, phase-appropriate delivery, and endpoints that capture both durable closure and scar quality.

## Glossary

**4EBP1**: eukaryotic translation initiation factor 4E-binding protein 1
**ACKR3**: atypical chemokine receptor 3
**ADNVs**: apple-derived nanovesicles
**ADSC**: adipose-derived stem cell
**ADSC-EVs**: extracellular vesicles from adipose-derived stem cells
**AKT**: protein kinase B
**AKT1**: AKT serine/threonine kinase 1
**AREG**: amphiregulin
**ARG1**: arginase 1
**ATG16L1**: autophagy-related 16-like 1
**BAX**: BCL2-associated X protein
**CCL2**: C-C motif chemokine ligand 2
**CCNE2**: cyclin E2
**CSF1**: colony-stimulating factor 1
**CXCL2**: C-X-C motif chemokine ligand 2
**CXCL12**: C-X-C motif chemokine ligand 12
**CXCR4**: C-X-C motif chemokine receptor 4
**CXCR7**: C-X-C chemokine receptor type 7
**DPC-EVs**: extracellular vesicles from dermal papilla cells
**ECM**: extracellular matrix
**ERK**: extracellular signal-regulated kinase
**ERK1/2**: extracellular signal-regulated kinases 1/2
**EVs**: extracellular vesicles
**GSDMD**: gasdermin D
**HSP72**: heat shock protein 72
**HSP90**: heat shock protein 90
**hUCMSC-EVs**: extracellular vesicles from human umbilical cord mesenchymal stromal cells
**IL**: interleukin
**JAK**: Janus kinase
**JAK2**: Janus kinase 2
**KLF4**: Krüppel-like factor 4
**LATS2**: large tumor suppressor kinase 2
**LPS**: lipopolysaccharide
**LRP1**: low-density lipoprotein receptor-related protein 1
**MALAT1**: metastasis-associated lung adenocarcinoma transcript 1
**MAPK**: mitogen-activated protein kinase
**MFG**-**E8**: milk fat globule-EGF factor 8
**MISEV 2023**: Minimal Information for Studies of Extracellular Vesicles 2023
MMT: macrophage-to-myofibroblast transition
MRC1: mannose receptor C-type 1
**MSC**: mesenchymal stromal cell
**MSC-EVs**: extracellular vesicles from mesenchymal stromal cells
**mTOR**: mechanistic target of rapamycin
**NF-κB**: nuclear factor kappa B
**NLRP3**: NLR family pyrin domain containing 3
**PDGF**: platelet-derived growth factor
**PI3K**: phosphoinositide 3-kinase
**PTEN**: phosphatase and tensin homolog
**ROCK**: Rho-associated kinase
**ROS**: reactive oxygen species
**SFL-3D**: self-feeder layer three-dimensional culture
**SIRT1**: sirtuin 1
**SMAD**: SMAD family proteins
**SOCS3**: suppressor of cytokine signaling 3
**STAT**: signal transducer and activator of transcription
**STAT3**: signal transducer and activator of transcription 3
**TAK1**: transforming growth factor-beta-activated kinase 1
**tdDPC-EVs**: extracellular vesicles from three-dimensional dermal papilla cells
**TGF-β**: transforming growth factor-beta
**TGF-β1**: transforming growth factor-beta 1
**TGFBR1**: transforming growth factor-beta receptor 1
**TGFBR2**: transforming growth factor-beta receptor 2
**VEGFA**: vascular endothelial growth factor A
**YAP/TAZ**: Yes-associated protein / transcriptional coactivator with PDZ-binding motif.

## References

[B1] Amuso V. M. Haas M. R. Cooper P. O. Chatterjee R. Hafiz S. Salameh S. (2025). Fibroblast-mediated macrophage recruitment supports acute wound healing. J. Invest Dermatol. 145 (7), 1781–1797.e8. 10.1016/j.jid.2024.10.609 39581458 PMC12095619

[B2] Andrews J. R. Kim Y. Horjeti E. Arafa A. Gunn H. De Bruycker A. (2025). PSMA+ extracellular vesicles are a biomarker for SABR in oligorecurrent prostate cancer: analysis from the STOMP-Like and ORIOLE trial cohorts. Clin. Cancer Res. 31 (6), 1142–1149. 10.1158/1078-0432.CCR-24-3027 PMC11911805 39820657

[B3] Aran D. Looney A. P. Liu L. Wu E. Fong V. Hsu A. (2019). Reference-based analysis of lung single-cell sequencing reveals a transitional profibrotic macrophage. Nat. Immunol. 20 (2), 163–172. 10.1038/s41590-018-0276-y 30643263 PMC6340744

[B4] Bhandari R. Yang H. Kosarek N. N. Smith A. E. Garlick J. A. Hinchcliff M. (2023). Human dermal fibroblast-derived exosomes induce macrophage activation in systemic sclerosis. Rheumatol. Oxf. 62 (Si), Si114–Si124. 10.1093/rheumatology/keac453 35946522 PMC9910573

[B5] Burridge K. Monaghan-Benson E. Graham D. M. (2019). Mechanotransduction: from the cell surface to the nucleus via RhoA. Philos. Trans. R. Soc. Lond B Biol. Sci. 374 (1779), 20180229. 10.1098/rstb.2018.0229 31431179 PMC6627015

[B6] Chandran N. S. Bhupendrabhai M. N. Tan T. T. Zhang B. Lim S. K. Choo A. B. H. (2025). A phase 1, open-label study to determine safety and tolerability of the topical application of mesenchymal stem/stromal cell (MSC) exosome ointment to treat psoriasis in healthy volunteers. Cytotherapy 27 (5), 633–641. 10.1016/j.jcyt.2025.01.007 39918488

[B7] Chen J. Zhou R. Liang Y. Fu X. Wang D. Wang C. (2019). Blockade of lncRNA-ASLNCS5088-enriched exosome generation in M2 macrophages by GW4869 dampens the effect of M2 macrophages on orchestrating fibroblast activation. Faseb J. 33 (11), 12200–12212. 10.1096/fj.201901610 31373848 PMC6902732

[B8] Chen J. Yu W. Xiao C. Su N. Han Y. Zhai L. (2023). Exosome from adipose-derived mesenchymal stem cells attenuates scar formation through microRNA-181a/SIRT1 axis. Arch. Biochem. Biophys. 746, 109733. 10.1016/j.abb.2023.109733 37652148

[B9] Chen C. Yang J. Shang R. Tang Y. Cai X. Chen Y. (2025). Orchestration of macrophage polarization dynamics by fibroblast-secreted exosomes during skin wound healing. J. Invest Dermatol. 145 (1), 171–184.e6. 10.1016/j.jid.2024.05.007 38838771

[B10] Cheng P. Li S. Chen H. (2021). Macrophages in lung injury, repair, and fibrosis. Cells 10 (2), 436. 10.3390/cells10020436 33670759 PMC7923175

[B11] Cho K. A. Kim H. J. Choi S. Woo S. Y. Roh J. Y. (2025). Semaphorin7A-expressing mast cell exosomes promote wound healing. Exp. Cell Res. 452 (2), 114771. 10.1016/j.yexcr.2025.114771 40992568

[B12] Cook K. Li H. (2025). Advancing extracellular vesicle production: improving physiological relevance and yield with 3D cell culture. Nanoscale 17 (25), 15110–15131. 10.1039/d5nr00707k 40512034

[B13] Cui H. S. Kim D. H. Joo S. Y. Cho Y. S. Kim J. B. Seo C. H. (2022). Exosomes derived from human hypertrophic scar fibroblasts induces smad and TAK1 signaling in normal dermal fibroblasts. Arch. Biochem. Biophys. 722, 109215. 10.1016/j.abb.2022.109215 35430216

[B14] Dai S. Xu M. Pang Q. Sun J. Lin X. Chu X. (2024). Hypoxia macrophage-derived exosomal miR-26b-5p targeting PTEN promotes the development of keloids. Burns Trauma 12, tkad036. 10.1093/burnst/tkad036 PMC10905499 38434721

[B15] Desmoulière A. Redard M. Darby I. Gabbiani G. (1995). Apoptosis mediates the decrease in cellularity during the transition between granulation tissue and scar. Am. J. Pathol. 146 (1), 56–66. 7856739 PMC1870783

[B16] Di X. Gao X. Peng L. Ai J. Jin X. Qi S. (2023). Cellular mechanotransduction in health and diseases: from molecular mechanism to therapeutic targets. Signal Transduct. Target Ther. 8 (1), 282. 10.1038/s41392-023-01501-9 37518181 PMC10387486

[B17] Ebrahimi F. Kumari A. Ghadami S. Al Abdullah S. Dellinger K. (2024). The potential for extracellular vesicles in nanomedicine: a review of recent advancements and challenges ahead. Adv. Biol. (Weinh). 9, e2400623. 10.1002/adbi.202400623 PMC12206938 39739455

[B18] Eerdekens H. Pirlet E. Willems S. Bronckaers A. Pincela Lins P. M. (2025). Extracellular vesicles: innovative cell-free solutions for wound repair. Front. Bioeng. Biotechnol. 13, 1571461. 10.3389/fbioe.2025.1571461 PMC12003306 40248643

[B19] Ferrer R. A. Saalbach A. Grünwedel M. Lohmann N. Forstreuter I. Saupe S. (2017). Dermal fibroblasts promote alternative macrophage activation improving impaired wound healing. J. Invest Dermatol. 137 (4), 941–950. 10.1016/j.jid.2016.11.035 28017830

[B20] Foster D. S. Januszyk M. Yost K. E. Chinta M. S. Gulati G. S. Nguyen A. T. (2021). Integrated spatial multiomics reveals fibroblast fate during tissue repair. Proc. Natl. Acad. Sci. U. S. A. 118 (41), e2110025118. 10.1073/pnas.2110025118 PMC8521719 34620713

[B21] Gorgoulis V. Adams P. D. Alimonti A. Bennett D. C. Bischof O. Bishop C. (2019). Cellular senescence: defining a path forward. Cell. 179 (4), 813–827. 10.1016/j.cell.2019.10.005 31675495

[B22] Han L. Song Y. Tong L. Sun J. Zhang X. Chen S. (2025). Extracellular vesicle protein panel enables early lung cancer detection in a large clinical cohort. J. Extracell. Vesicles 14 (8), e70129. 10.1002/jev2.70129 PMC12326191 40766981

[B23] Hassanshahi A. Moradzad M. Ghalamkari S. Fadaei M. Cowin A. J. Hassanshahi M. (2022). Macrophage-mediated inflammation in skin wound healing. Cells 11 (19), 2953. 10.3390/cells11192953 PMC9564023 36230913

[B24] Hesketh M. Sahin K. B. West Z. E. Murray R. Z. (2017). Macrophage phenotypes regulate scar formation and chronic wound healing. Int. J. Mol. Sci. 18 (7), 1545. 10.3390/ijms18071545 28714933 PMC5536033

[B25] Hsu H. H. Wang A. Y. L. Loh C. Y. Y. Pai A. A. Kao H. K. (2022). Therapeutic potential of exosomes derived from diabetic adipose stem cells in cutaneous wound healing of Db/Db mice. Pharmaceutics 14 (6), 1206. 10.3390/pharmaceutics14061206 PMC9227821 35745779

[B26] Hyun S. Choi H. Sub Y. Hong D. Ahn S. H. Choi K. (2025). Safety and anti-inflammatory effects of engineered extracellular vesicles (ILB-202) for NF-κB inhibition: a double-blind, randomized, placebo-controlled phase 1 trial. J. Extracell. Vesicles 14 (9), e70141. 10.1002/jev2.70141 PMC12465005 41002119

[B27] Jarnagin H. C. Parvizi R. Gong Z. Gedert R. Xing X. Tsoi L. A. C. (2026). Multimodal analyses of early, untreated systemic sclerosis skin identify a proinflammatory vascular niche of macrophage-fibroblast signaling. JCI Insight 11 (3), e198954. 10.1172/jci.insight.198954 PMC12893110 41411065

[B28] Jiang X. Ma J. Xue K. Chen J. Zhang Y. Zhang G. (2024). Highly bioactive MXene-M2-Exosome nanocomposites promote angiogenic diabetic wound repair through reconstructing high glucose-derived immune inhibition. ACS Nano 18 (5), 4269–4286. 10.1021/acsnano.3c09721 38270104

[B29] Johnson J. Law S. Q. K. Shojaee M. Hall A. S. Bhuiyan S. Lim M. B. L. (2023). First-in-human clinical trial of allogeneic, platelet-derived extracellular vesicles as a potential therapeutic for delayed wound healing. J. Extracell. Vesicles 12 (7), e12332. 10.1002/jev2.12332 37353884 PMC10290200

[B30] Kang D. Wang X. Chen W. Mao L. Zhang W. Shi Y. (2024). Epidermal stem cell-derived exosomes improve wound healing by promoting the proliferation and migration of human skin fibroblasts. Burns Trauma 12, tkae047. 10.1093/burnst/tkae047 PMC11647520 39687464

[B31] Kim S. Kim Y. Hyun Y. S. Choi H. Kim S. Y. Kim T. G. (2021). Exosomes from human cord blood plasma accelerate cutaneous wound healing by promoting fibroblast function, angiogenesis, and M2 macrophage differentiation. Biomater. Sci. 9 (8), 3028–3039. 10.1039/d0bm01801e 33657200

[B32] Kishta M. S. Hafez A. M. Hydara T. Hamed Z. Bahr M. M. Shamaa A. A. (2025). The transforming role of wharton's jelly mesenchymal stem cell-derived exosomes for diabetic foot ulcer healing: a randomized controlled clinical trial. Stem Cell Res. Ther. 16 (1), 559. 10.1186/s13287-025-04690-y 41084065 PMC12519741

[B33] Kuang L. Zhang C. Li B. Deng H. Chen R. Li G. (2023). Human keratinocyte-derived exosomal MALAT1 promotes diabetic wound healing by upregulating MFGE8 via microRNA-1914-3p. Int. J. Nanomedicine 18, 949–970. 10.2147/IJN.S399785 PMC9961177 36852184

[B34] Las Heras K. Royo F. Garcia-Vallicrosa C. Igartua M. Santos-Vizcaino E. Falcon-Perez J. M. (2022). Extracellular vesicles from hair follicle-derived mesenchymal stromal cells: isolation, characterization and therapeutic potential for chronic wound healing. Stem Cell Res. Ther. 13 (1), 147. 10.1186/s13287-022-02824-0 35395929 PMC8994406

[B35] Lener T. Gimona M. Aigner L. Börger V. Buzas E. Camussi G. (2015). Applying extracellular vesicles based therapeutics in clinical trials - an ISEV position paper. J. Extracell. Vesicles 4, 30087. 10.3402/jev.v4.30087 26725829 PMC4698466

[B36] Li Y. Zhang J. Shi J. Liu K. Wang X. Jia Y. (2021). Exosomes derived from human adipose mesenchymal stem cells attenuate hypertrophic scar fibrosis by miR-192-5p/IL-17RA/Smad axis. Stem Cell Res. Ther. 12 (1), 221. 10.1186/s13287-021-02290-0 33789737 PMC8010995

[B37] Li C. An Y. Sun Y. Yang F. Xu Q. Wang Z. (2022). Adipose mesenchymal stem cell-derived exosomes promote wound healing through the WNT/β-catenin signaling pathway in dermal fibroblasts. Stem Cell Rev. Rep. 18 (6), 2059–2073. 10.1007/s12015-022-10378-0 PMC9391246 35471485

[B38] Li Z. Rius Rigau A. Xie W. Huang L. Ye W. Li Y. N. (2025a). Spatial multiomics decipher fibroblast-macrophage dynamics in systemic sclerosis. Ann. Rheum. Dis. 84 (7), 1231–1245. 10.1016/j.ard.2025.04.025 40410053

[B39] Li M. Huang H. Wei X. Li H. Li J. Xie B. (2025b). Clinical investigation on nebulized human umbilical cord MSC-Derived extracellular vesicles for pulmonary fibrosis treatment. Signal Transduct. Target Ther. 10 (1), 179. 10.1038/s41392-025-02262-3 PMC12134356 40461474

[B40] Liang Q. Zhou D. Ge X. Song P. Chu W. Xu J. (2024). Exosomes from adipose-derived mesenchymal stem cell improve diabetic wound healing and inhibit fibrosis via miR-128-1-5p/TGF-β1/Smad axis. Mol. Cell Endocrinol. 588, 112213. 10.1016/j.mce.2024.112213 38556162

[B41] Lin Y. Xu Z. (2020). Fibroblast senescence in idiopathic pulmonary fibrosis. Front. Cell Dev. Biol. 8, 593283. 10.3389/fcell.2020.593283 33324646 PMC7723977

[B42] Liu C. Khairullina L. Qin Y. Zhang Y. Xiao Z. (2024a). Adipose stem cell exosomes promote mitochondrial autophagy through the PI3K/AKT/mTOR pathway to alleviate keloids. Stem Cell Res. Ther. 15 (1), 305. 10.1186/s13287-024-03928-5 PMC11403874 39278919

[B43] Liu B. Xian Y. Chen X. Shi Y. Dong J. Yang L. (2024b). Inflammatory fibroblast-like Synoviocyte-Derived exosomes aggravate osteoarthritis via enhancing macrophage glycolysis. Adv. Sci. (Weinh) 11 (14), e2307338. 10.1002/advs.202307338 PMC11005727 38342630

[B44] Liu Z. Bian X. Luo L. Björklund Å. K. Li L. Zhang L. (2025a). Spatiotemporal single-cell roadmap of human skin wound healing. Cell Stem Cell 32 (3), 479–498.e8. 10.1016/j.stem.2024.11.013 39729995

[B45] Liu Z. Gu J. Gao Y. Hu H. Jiang H. (2025b). ADSC-Derived exosomes mitigate radiation-induced skin injury by reducing oxidative stress, inflammation and cell death. Front. Public Health 13, 1603431. 10.3389/fpubh.2025.1603431 PMC12116344 40438040

[B46] Lu B. Jin J. Jin W. Yuan X. Jin Z. (2026). Extracellular vesicle-mediated cell-cell communication in keloids and hypertrophic scars: mechanisms, methodological caveats, and therapeutic perspectives. Front. Cell Dev. Biol. 14, 1818463. 10.3389/fcell.2026.1818463 42306298 PMC13265475

[B47] Ma F. Li Y. Jia L. Han Y. Cheng J. Li H. (2012). Macrophage-stimulated cardiac fibroblast production of IL-6 is essential for TGF β/Smad activation and cardiac fibrosis induced by angiotensin II. PLoS One 7 (5), e35144. 10.1371/journal.pone.0035144 22574112 PMC3344835

[B48] Ma J. Zhang Z. Wang Y. Shen H. (2022). Investigation of miR-126-3p loaded on adipose stem cell-derived exosomes for wound healing of full-thickness skin defects. Exp. Dermatol 31 (3), 362–374. 10.1111/exd.14480 34694648

[B49] Ma Y. Liu Z. Miao L. Jiang X. Ruan H. Xuan R. (2023). Mechanisms underlying pathological scarring by fibroblasts during wound healing. Int. Wound J. 20 (6), 2190–2206. 10.1111/iwj.14097 36726192 PMC10333014

[B50] Ma X. Wei W. Zeng H. Dong M. (2025). 3D-cultured DFAT spheroid-derived exosomes promote aged wound healing via modulation of the NF-κB/Serpine1 pathway. Tissue Cell 97, 103049. 10.1016/j.tice.2025.103049 40712434

[B51] Meng S. Wei Q. Chen S. Liu X. Cui S. Huang Q. (2024). MiR-141-3p-Functionalized exosomes loaded in dissolvable microneedle arrays for hypertrophic scar treatment. Small 20 (8), e2305374. 10.1002/smll.202305374 37724002

[B52] Minutti C. M. Modak R. V. Macdonald F. Li F. Smyth D. J. Dorward D. A. (2019). A macrophage-pericyte axis directs tissue restoration via amphiregulin-induced transforming growth factor beta activation. Immunity 50 (3), 645–654.e6. 10.1016/j.immuni.2019.01.008 30770250 PMC6436929

[B53] Murray P. J. Allen J. E. Biswas S. K. Fisher E. A. Gilroy D. W. Goerdt S. (2014). Macrophage activation and polarization: nomenclature and experimental guidelines. Immunity 41 (1), 14–20. 10.1016/j.immuni.2014.06.008 25035950 PMC4123412

[B54] Nguyen V. V. T. Witwer K. W. Verhaar M. C. Strunk D. van Balkom B. W. M. (2020). Functional assays to assess the therapeutic potential of extracellular vesicles. J. Extracell. Vesicles 10 (1), e12033. 10.1002/jev2.12033 33708360 PMC7890556

[B55] Reiner A. T. Witwer K. W. van Balkom B. W. M. de Beer J. Brodie C. Corteling R. L. (2017). Concise review: developing best-practice models for the therapeutic use of extracellular vesicles. Stem Cells Transl. Med. 6 (8), 1730–1739. 10.1002/sctm.17-0055 28714557 PMC5689784

[B56] Ren S. Chen J. Guo J. Liu Y. Xiong H. Jing B. (2022). Exosomes from adipose stem cells promote diabetic wound healing through the eHSP90/LRP1/AKT axis. Cells 11 (20), 3229. 10.3390/cells11203229 PMC9600018 36291096

[B57] Sadeghi Moghaddam Bijari A. Alijanianzadeh M. Keshmiri Neghab H. Soheilifar M. H. (2025). A mini-review on fibroblast-derived exosomes as wound healing stimulators. Int. J. Hematol. Oncol. Stem Cell Res. 19 (3), 237–247. 10.18502/ijhoscr.v19i3.19287 PMC12630532 41281797

[B58] Saeki N. Imai Y. (2020). Reprogramming of synovial macrophage metabolism by synovial fibroblasts under inflammatory conditions. Cell Commun. Signal 18 (1), 188. 10.1186/s12964-020-00678-8 33256735 PMC7708128

[B59] Shang Y. Li M. Zhang L. Han C. Shen K. Wang K. (2024). Exosomes derived from mouse vibrissa dermal papilla cells promote hair follicle regeneration during wound healing by activating Wnt/β-catenin signaling pathway. J. Nanobiotechnology 22 (1), 425. 10.1186/s12951-024-02689-w 39030543 PMC11264511

[B60] Shao Z. Xu J. Wang X. Zhou Y. Wang Y. Li Y. (2024). Exosomes derived from adipose tissues accelerate fibroblasts and keratinocytes proliferation and cutaneous wound healing via miR-92a/Hippo-YAP axis. J. Physiol. Biochem. 80 (1), 189–204. 10.1007/s13105-023-00996-8 38041784

[B61] Shi M. Zhang L. Bi F. Ma X. (2024). Exosomes derived from M2 macrophages promote fibroblast autophagy to contribute to hypertrophic scar formation via CXCL2/CXCR7/mTOR pathway. Hum. Exp. Toxicol. 43, 9603271241303320. 10.1177/09603271241303320 39557042

[B62] Shi S. Ou X. Wang Q. Zhang L. (2025). Macrophage-derived extracellular vesicles: a novel therapeutic alternative for diabetic wound. Int. J. Nanomedicine 20, 5763–5777. 10.2147/IJN.S518655 PMC12060905 40343196

[B63] Shim J. Oh S. J. Yeo E. Park J. H. Bae J. H. Kim S. H. (2022). Integrated analysis of single-cell and spatial transcriptomics in keloids: highlights on fibrovascular interactions in keloid pathogenesis. J. Invest Dermatol. 142 (8), 2128–2139.e11. 10.1016/j.jid.2022.01.017 35123990

[B64] Shook B. A. Wasko R. R. Rivera-Gonzalez G. C. Salazar-Gatzimas E. López-Giráldez F. Dash B. C. (2018). Myofibroblast proliferation and heterogeneity are supported by macrophages during skin repair. Science 362 (6417), eaar2971. 10.1126/science.aar2971 30467144 PMC6684198

[B65] Song P. Liang Q. Ge X. Zhou D. Yuan M. Chu W. (2025a). Adipose-derived stem cell exosomes promote scar-free healing of diabetic wounds via miR-204-5p/TGF-β1/Smad pathway. Stem Cells Int. 2025, 6344844. 10.1155/sci/6344844 PMC11865461 40018015

[B66] Song S. Xiang R. Chen S. Wu J. Chen W. Li X. (2025b). Saliva-derived exosomes regulate fibroblast metabolic reprogramming in skin wound healing. Front. Cell Dev. Biol. 13, 1606716. 10.3389/fcell.2025.1606716 40772233 PMC12325227

[B67] Steele L. Olabi B. Roberts K. Mazin P. V. Koplev S. Tudor C. (2025). A single-cell and spatial genomics atlas of human skin fibroblasts reveals shared disease-related fibroblast subtypes across tissues. Nat. Immunol. 26 (10), 1807–1820. 10.1038/s41590-025-02267-8 PMC12479362 40993240

[B68] Stierli S. Salas-Bastos A. Micheli S. Ballwein I. Kelemen A. Lehmann J. (2026). A peripheral glial niche orchestrates the early stages of skin wound healing. Cell Stem Cell 33 (2), 272–288.e10. 10.1016/j.stem.2025.12.015 41512873

[B69] Storci G. De Felice F. Ricci F. Santi S. Messelodi D. Bertuccio S. N. (2024). CAR+ extracellular vesicles predict ICANS in patients with B cell lymphomas treated with CD19-directed CAR T cells. J. Clin. Invest 134 (14), e173096. 10.1172/JCI173096 PMC11245152 38833312

[B70] Su Y. Huang Z. Chen Y. Deng J. Huang Y. Xiong W. (2025). Exosomes from miR-21-5p-modified adipose-derived stem cells promote wound healing by regulating M2 macrophage polarization in a rodent model of pressure ulcer. J. Mol. Histol. 56 (3), 135. 10.1007/s10735-025-10407-5 40249566

[B71] Sun D. S. Chang H. H. (2024). Extracellular vesicles: function, resilience, biomarker, bioengineering, and clinical implications. Tzu Chi Med. J. 36 (3), 251–259. 10.4103/tcmj.tcmj_28_24 38993825 PMC11236075

[B72] Sun N. N. Zhang Y. Huang W. H. Zheng B. J. Jin S. Y. Li X. (2021). Macrophage exosomes transfer angiotensin II type 1 receptor to lung fibroblasts mediating bleomycin-induced pulmonary fibrosis. Chin. Med. J. Engl. 134 (18), 2175–2185. 10.1097/cm9.0000000000001605 34483252 PMC8478379

[B73] Sun H. Dong J. Fu Z. Lu X. Chen X. Lei H. (2024). TSG6-Exo@CS/GP attenuates endometrium fibrosis by inhibiting macrophage activation in a murine IUA model. Adv. Mater 36 (21), e2308921. 10.1002/adma.202308921 38588501

[B74] Talbott H. E. Mascharak S. Griffin M. Wan D. C. Longaker M. T. (2022). Wound healing, fibroblast heterogeneity, and fibrosis. Cell Stem Cell 29 (8), 1161–1180. 10.1016/j.stem.2022.07.006 PMC9357250 35931028

[B75] Teng L. Maqsood M. Zhu M. Zhou Y. Kang M. Zhou J. (2022). Exosomes derived from human umbilical cord mesenchymal stem cells accelerate diabetic wound healing via promoting M2 macrophage polarization, angiogenesis, and collagen deposition. Int. J. Mol. Sci. 23 (18), 10421. 10.3390/ijms231810421 36142334 PMC9498995

[B76] Theocharidis G. Thomas B. E. Sarkar D. Mumme H. L. Pilcher W. J. R. Dwivedi B. (2022). Single cell transcriptomic landscape of diabetic foot ulcers. Nat. Commun. 13 (1), 181. 10.1038/s41467-021-27801-8 PMC8748704 35013299

[B77] Tian C. Xu H. Li C. Gao J. Zhang H. Wang P. (2025). ADSC exosomes improve high glucose induced fibroblast oxidative stress injury and accelerate DFU wound healing via regulating Keap1/Nrf2 axis. Cell Signal 134, 111936. 10.1016/j.cellsig.2025.111936 40505843

[B78] Torres Á. Muñoz K. Nahuelpán Y. Ap R. S. Mendoza P. Jara C. (2020). Intraglomerular monocyte/macrophage infiltration and macrophage-myofibroblast transition during diabetic nephropathy is regulated by the A(2B) adenosine receptor. Cells 9 (4), 1051. 10.3390/cells9041051 32340145 PMC7226348

[B79] Trentini M. Zanolla I. Zanotti F. Tiengo E. Licastro D. Dal Monego S. (2022). Apple derived exosomes improve collagen type I production and decrease MMPs during aging of the skin through downregulation of the NF-κB pathway as mode of action. Cells 11 (24), 3950. 10.3390/cells11243950 36552714 PMC9776931

[B80] Turzańska K. Adesanya O. Rajagopal A. Pryce M. T. Fitzgerald Hughes D. (2023). Improving the management and treatment of diabetic foot infection: challenges and research opportunities. Int. J. Mol. Sci. 24 (4), 3913. 10.3390/ijms24043913 36835330 PMC9959562

[B81] Ueshima E. Fujimori M. Kodama H. Felsen D. Chen J. Durack J. C. (2019). Macrophage-secreted TGF-β(1) contributes to fibroblast activation and ureteral stricture after ablation injury. Am. J. Physiol. Ren. Physiol. 317 (7), F52–f64. 10.1152/ajprenal.00260.2018 31017012 PMC6692725

[B82] Vanherle S. Guns J. Loix M. Mingneau F. Dierckx T. Wouters F. (2023). Extracellular vesicle-associated cholesterol supports the regenerative functions of macrophages in the brain. J. Extracell. Vesicles 12 (12), e12394. 10.1002/jev2.12394 PMC10733568 38124258

[B83] Vierhout M. Ayoub A. Naiel S. Yazdanshenas P. Revill S. D. Reihani A. (2021). Monocyte and macrophage derived myofibroblasts: is it fate? A review of the current evidence. Wound Repair Regen. 29 (4), 548–562. 10.1111/wrr.12946 34107123

[B84] Vollmers A. C. Wu S. Z. Altieri A. McCartney E. E. Bender H. Davidson C. D. (2026). Reciprocal regulation of fibroblast-macrophage equilibrium governs skin integrity. Nat. Immunol. 27 (4), 700–714. 10.1038/s41590-026-02434-5 PMC13043295 41760904

[B85] Wang Y. Jing L. Lei X. Ma Z. Li B. Shi Y. (2023a). Umbilical cord mesenchymal stem cell-derived apoptotic extracellular vesicles ameliorate cutaneous wound healing in type 2 diabetic mice via macrophage pyroptosis inhibition. Stem Cell Res. Ther. 14 (1), 257. 10.1186/s13287-023-03490-6 37726853 PMC10510296

[B86] Wang Y. Shen K. Sun Y. Cao P. Zhang J. Zhang W. (2023b). Extracellular vesicles from 3D cultured dermal papilla cells improve wound healing via Krüppel-like factor 4/vascular endothelial growth factor A -driven angiogenesis. Burns Trauma 11, tkad034. 10.1093/burnst/tkad034 37908562 PMC10615254

[B87] Wang S. Wu J. Ren K. Zhang Y. Gao F. Chen Y. (2025a). Platelet-rich plasma-derived exosome-encapsulated hydrogels accelerate diabetic wound healing by inhibiting fibroblast ferroptosis. ACS Appl. Mater Interfaces 17 (19), 27923–27936. 10.1021/acsami.5c02705 40315047

[B88] Wang Y. Kong Y. Du J. Qi L. Liu M. Xie S. (2025b). Injection of human umbilical cord mesenchymal stem cells exosomes for the treatment of knee osteoarthritis: from preclinical to clinical research. J. Transl. Med. 23 (1), 641. 10.1186/s12967-025-06623-y PMC12153132 40500748

[B89] Wang Y. Zhao L. Ma H. Shi A. Cao P. Cai F. (2026a). Self-organizing three-dimensional dermal papilla cell spheroids yield therapeutic extracellular vesicles that target hypertrophic scar regression via the miR-26a-5p/CCNE2 axis. Burns Trauma 14, tkaf048. 10.1093/burnst/tkaf048 PMC13345373 42421776

[B90] Wang Y. Hu Z. Shi A. Cao P. Zhao L. Di X. (2026b). SFL-3D-Cultured adipose mesenchymal cell-derived extracellular vesicles promote diabetic wound healing by alleviating microvascular endothelial senescence via PI3K/AKT/mTOR/4EBP1-Mediated cap-dependent translation. Burns and Trauma. 14, tkag042. 10.1093/burnst/tkag042 PMC13571603 42733811

[B91] Welsh J. A. Goberdhan D. C. I. O'Driscoll L. Buzas E. I. Blenkiron C. Bussolati B. (2024). Minimal information for studies of extracellular vesicles (MISEV2023): from basic to advanced approaches. J. Extracell. Vesicles 13 (2), e12404. 10.1002/jev2.12404 38326288 PMC10850029

[B92] Wolf M. Poupardin R. W. Ebner-Peking P. Andrade A. C. Blöchl C. Obermayer A. (2022). A functional Corona around extracellular vesicles enhances angiogenesis, skin regeneration and immunomodulation. J. Extracell. Vesicles 11 (4), e12207. 10.1002/jev2.12207 PMC8994701 35398993

[B93] Xia W. Li M. Jiang X. Huang X. Gu S. Ye J. (2022). Young fibroblast-derived exosomal microRNA-125b transfers beneficial effects on aged cutaneous wound healing. J. Nanobiotechnology 20 (1), 144. 10.1186/s12951-022-01348-2 35305652 PMC9744129

[B94] Xiao Y. Qian J. Deng X. Zhang H. Wang J. Luo Z. (2024). Macrophages regulate healing-associated fibroblasts in diabetic wound. Mol. Biol. Rep. 51 (1), 203. 10.1007/s11033-023-09100-1 38270651 PMC10811177

[B95] Xie Y. Yu L. Cheng Z. Peng Y. Cao Z. Chen B. (2022). SHED-Derived exosomes promote LPS-Induced wound healing with less itching by stimulating macrophage autophagy. J. Nanobiotechnology 20 (1), 239. 10.1186/s12951-022-01446-1 35597946 PMC9124392

[B96] Xiong Y. Chang Y. Hao J. Zhang C. Yang F. Wang Z. (2021). Eplerenone attenuates fibrosis in the contralateral kidney of UUO rats by preventing macrophage-to-myofibroblast transition. Front. Pharmacol. 12, 620433. 10.3389/fphar.2021.620433 33716747 PMC7943730

[B97] Xiong Q. H. Zhao L. Wan G. Q. Hu Y. G. Li X. L. (2023). Engineered BMSCs-Derived exosomal miR-542-3p promotes cutaneous wound healing. Endocr. Metab. Immune Disord. Drug Targets 23 (3), 336–346. 10.2174/1871530322666220523151713 35616673

[B98] Xu X. Gu S. Huang X. Ren J. Gu Y. Wei C. (2020). The role of macrophages in the formation of hypertrophic scars and keloids. Burns Trauma 8, tkaa006. 10.1093/burnst/tkaa006 32341919 PMC7175772

[B99] Xu C. Zhang H. Yang C. Wang Y. Wang K. Wang R. (2024). miR-125b-5p delivered by adipose-derived stem cell exosomes alleviates hypertrophic scarring by suppressing Smad2. Burns Trauma 12, tkad064. 10.1093/burnst/tkad064 38765787 PMC11102599

[B100] Xu Z. Ni T. Zhang Q. Sun X. Zhao L. Lin J. (2025). Exosomes derived from fibroblasts in DFUs delay wound healing by delivering miR-93-5p to target macrophage ATG16L1. Biochim. Biophys. Acta Mol. Basis Dis. 1871 (3), 167640. 10.1016/j.bbadis.2024.167640 39761761

[B101] Younesi F. S. Miller A. E. Barker T. H. Rossi F. M. V. Hinz B. (2024). Fibroblast and myofibroblast activation in normal tissue repair and fibrosis. Nat. Rev. Mol. Cell Biol. 25 (8), 617–638. 10.1038/s41580-024-00716-0 38589640

[B102] Yang F. Chang Y. Zhang C. Xiong Y. Wang X. Ma X. (2021). UUO induces lung fibrosis with macrophage-myofibroblast transition in rats. Int. Immunopharmacol. 93, 107396. 10.1016/j.intimp.2021.107396 33540244

[B103] Yang H. Xu H. Wang Z. Li X. Wang P. Cao X. (2023). Analysis of miR-203a-3p/SOCS3-mediated induction of M2 macrophage polarization to promote diabetic wound healing based on epidermal stem cell-derived exosomes. Diabetes Res. Clin. Pract. 197, 110573. 10.1016/j.diabres.2023.110573 36764461

[B104] Yang C. Zhang H. Zeng C. Tian C. Liu W. Chen Y. (2024). Exosomes from adipose-derived stem cells restore fibroblast function and accelerate diabetic wound healing. Heliyon 10 (1), e22802. 10.1016/j.heliyon.2023.e22802 38163237 PMC10755272

[B105] Yazicioglu S. Arslan T. S. Erdoğan Y. K. Ercan B. Arslan Y. E. Derkus B. (2026). M2-Macrophage-Derived extracellular vesicles-functionalized acellular dermal matrix as a new-generation immunoregulatory and angio-inductive construct for skin tissue engineering. Macromol. Biosci. 26 (1), e00500. 10.1002/mabi.202500500 41582295

[B106] Yin D. Shen G. (2024). Exosomes from adipose-derived stem cells regulate macrophage polarization and accelerate diabetic wound healing via the circ-Rps5/miR-124-3p axis. Immun. Inflamm. Dis. 12 (6), e1274. 10.1002/iid3.1274 38888351 PMC11184652

[B107] Yuan R. Dai X. Li Y. Li C. Liu L. (2021). Exosomes from miR-29a-modified adipose-derived mesenchymal stem cells reduce excessive scar formation by inhibiting TGF-β2/Smad3 signaling. Mol. Med. Rep. 24 (5), 758. 10.3892/mmr.2021.12398 34476508 PMC8436211

[B108] Zeng C. Tan Y. Jiao Z. Hu S. Tang S. Shi Q. (2026). A multicenter randomized controlled trial of intrapleural perfusion of methotrexate-loaded tumor cell-derived microparticles combined with systemic therapy for malignant pleural effusion. Int. J. Cancer 158 (1), 172–181. 10.1002/ijc.70094 40818045

[B109] Zhang Y. Pan Y. Liu Y. Li X. Tang L. Duan M. (2021a). Exosomes derived from human umbilical cord blood mesenchymal stem cells stimulate regenerative wound healing via transforming growth factor-β receptor inhibition. Stem Cell Res. Ther. 12 (1), 434. 10.1186/s13287-021-02517-0 PMC8336384 34344478

[B110] Zhang Y. Yan J. Liu Y. Chen Z. Li X. Tang L. (2021b). Human amniotic fluid stem cell-derived exosomes as a novel cell-free therapy for cutaneous regeneration. Front. Cell Dev. Biol. 9, 685873. 10.3389/fcell.2021.685873 PMC8255501 34235150

[B111] Zhang H. Zang C. Zhao W. Zhang L. Liu R. Feng Z. (2023). Exosome derived from mesenchymal stem cells alleviates hypertrophic scar by inhibiting the fibroblasts via TNFSF-13/HSPG2 signaling pathway. Int. J. Nanomedicine 18, 7047–7063. 10.2147/IJN.S433510 38046235 PMC10693282

[B112] Zhang C. Xiao W. Wang H. Li L. Yang Y. Hao Y. (2024). Exosomes derived from mouse breast carcinoma cells facilitate diabetic wound healing. Tissue Eng. Regen. Med. 21 (4), 571–586. 10.1007/s13770-024-00629-1 38472732 PMC11087414

[B113] Zhao X. Kwan J. Y. Y. Yip K. Liu P. P. Liu F. F. (2020). Targeting metabolic dysregulation for fibrosis therapy. Nat. Rev. Drug Discov. 19 (1), 57–75. 10.1038/s41573-019-0040-5 31548636

[B114] Zhao W. Zhang R. Zang C. Zhang L. Zhao R. Li Q. (2022). Exosome derived from mesenchymal stem cells alleviates pathological scars by inhibiting the proliferation, migration and protein expression of fibroblasts via delivering miR-138-5p to target SIRT1. Int. J. Nanomedicine 17, 4023–4038. 10.2147/IJN.S377317 PMC9467851 36105616

[B115] Zhou M. Li Y. J. Tang Y. C. Hao X. Y. Xu W. J. Xiang D. X. (2022). Apoptotic bodies for advanced drug delivery and therapy. J. Control Release 351, 394–406. 10.1016/j.jconrel.2022.09.045 36167267

[B116] Zhu Z. Chen B. Peng L. Gao S. Guo J. Zhu X. (2021). Blockade of LINC01605-enriched exosome generation in M2 macrophages impairs M2 macrophage-induced proliferation, migration, and invasion of human dermal fibroblasts. Int. J. Immunopathol. Pharmacol. 35, 20587384211016724. 10.1177/20587384211016724 34011185 PMC8150463

